# Astrocyte heterogeneity reveals region-specific astrogenesis in the white matter

**DOI:** 10.1038/s41593-025-01878-6

**Published:** 2025-02-24

**Authors:** Riccardo Bocchi, Manja Thorwirth, Tatiana Simon-Ebert, Christina Koupourtidou, Solène Clavreul, Keegan Kolf, Patrizia Della Vecchia, Sara Bottes, Sebastian Jessberger, Jiafeng Zhou, Gulzar Wani, Gregor-Alexander Pilz, Jovica Ninkovic, Annalisa Buffo, Swetlana Sirko, Magdalena Götz, Judith Fischer-Sternjak

**Affiliations:** 1 Chair of Physiological Genomics, Biomedical Center (BMC), Faculty of Medicine, LMU Munich, Munich, Germany; 2 Institute of Stem Cell Research, Helmholtz Center Munich, German Research Center for Environmental Health, Neuherberg, Germany; 3 Chair of Cell Biology and Anatomy, Biomedical Center (BMC), Faculty of Medicine, LMU Munich, Munich, Germany; 4 Laboratory of Neural Plasticity, Brain Research Institute, University of Zurich, Zurich, Switzerland; 5 Department of Basic Neurosciences, University of Geneva, Geneva, Switzerland; 6 Department of Neuroscience Rita Levi-Montalcini, University of Turin, Turin, Italy; 7 Neuroscience Institute Cavalieri Ottolenghi, University of Turin, Orbassano, Italy; 8 Excellence Cluster of Systems Neurology (SyNergy), Munich, Germany; 9 Present Address: Department of Basic Neurosciences, University of Geneva, Geneva, Switzerland

**Keywords:** Astrocyte, Gliogenesis, Genetics of the nervous system, Molecular neuroscience

## Abstract

Astrocyte heterogeneity has been well explored, but our understanding of white matter (WM) astrocytes and their distinctions from gray matter (GM) astrocytes remains limited. Here, we compared astrocytes from cortical GM and WM/corpus callosum (WM/CC) using single-cell RNA sequencing and spatial transcriptomics of the murine forebrain. The comparison revealed similarities but also significant differences between WM and GM astrocytes, including cytoskeletal and metabolic hallmarks specific to WM astrocytes with molecular properties also shared with human WM astrocytes. When we compared murine astrocytes from two different WM regions, the cortex and cerebellum, we found that they exhibited distinct, region-specific molecular properties, with the cerebellum lacking, for example, a specific cluster of WM astrocytes expressing progenitor and proliferation genes. Functional experiments confirmed astrocyte proliferation in the WM/CC, but not in the cerebellar WM, suggesting that the WM/CC may be a source of continued astrogenesis.

## Abstract (web summary)

White matter (WM) astrocytes differ significantly from gray matter astrocytes, with WM astrocytes in the forebrain exhibiting unique proliferation capacity, which is absent in cerebellar WM, suggesting region-specific astrocyte generation.

## Main

Astrocytes represent a highly abundant cell type in the central nervous system (CNS)^1^, fulfilling important functions, including support of neuronal metabolism, synaptogenesis, neurotransmitter recycling and neuronal survival^2,3^. This prompts the question how homogeneous astrocytes are, or if subtypes dedicated to distinct functions may exist. The origins of the identification of astrocyte heterogeneity date back to Ramón y Cajal’s demonstration of multiple morphological variations^4^, as is also the case for protoplasmic astrocytes in the gray matter (GM) differing morphologically from fibrous astrocytes in the white matter (WM)^5–8^. However, astrocytes with different morphology and anatomical locations also differ molecularly and functionally across the CNS and in specific brain regions^3,9–14^. Surprisingly, a comprehensive molecular analysis of WM astrocytes from different brain regions is still lacking because regionalization and molecular features have been studied primarily in GM regions or combining GM and WM^15,16^.

Our recent single-cell RNA sequencing (scRNA-seq) analysis of diencephalic astrocytes revealed that some subtypes share gene expression with astrocytes from other regions, while others have region-specific hallmarks and therefore a more restricted spatial distribution^17^. In the same study, we also found that a subset of astrocytes displayed some degree of proliferation, supporting the idea of ongoing adult astrogenesis in the diencephalon, as later also reported in the dentate gyrus (DG)^18^. These findings raise the question of whether this proliferative capacity is unique to a subset of astrocytes in the diencephalon and the DG or if similar abilities exist in astrocytes from other brain regions.

Hence, we investigated single-cell gene expression in WM astrocytes across diverse regions (cortical WM/corpus callosum (WM/CC) and cerebellum) and species (mouse and human). Comparing their molecular features with GM astrocytes, we identified shared, region-specific and species-specific hallmarks. This analysis also highlighted a subgroup of astrocytes in the mouse WM/CC as a source of continued astrogenesis.

## Results

### Single-cell profiling reveals distinct WM and GM cell types

**Fig. 1:**
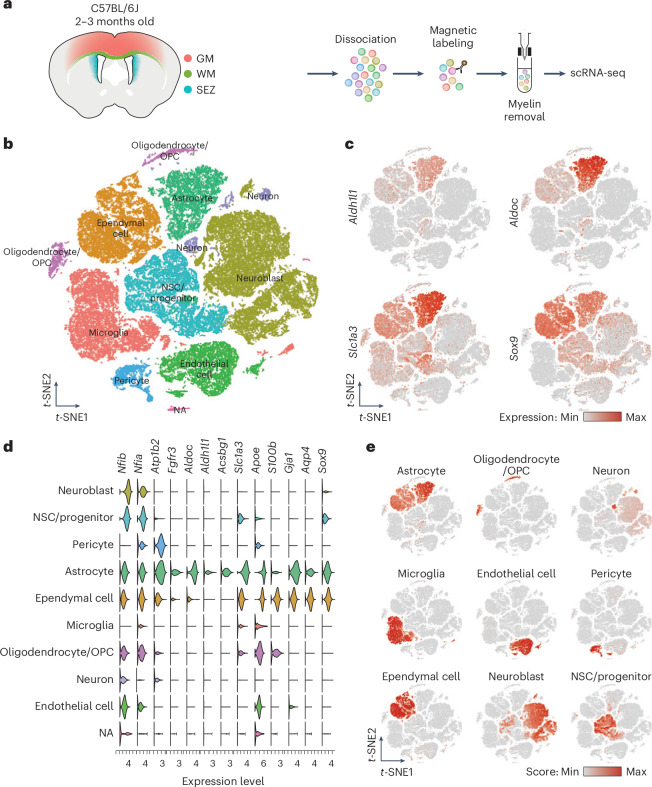
Isolation of single cells from cortical GM, WM and the SEZ. **a**, Schematic representation of the workflow used to dissect all three regions from 2–3-month-old C57BL/6J mice to generate the single-cell suspension for scRNA-seq. **b**, *t*-distributed stochastic neighbor embedding (*t*-SNE) visualization of scRNA-seq data from the three regions, with cells color-coded based on their cell types. **c**, *t*-SNE visualization showing expression levels (normalized) of common markers for astrocytes. **d**, Gene expression profiles obtained from scRNA-seq data, segregated according to the cell types identified in **b** and representing genes used to calculate the astrocyte score. **e**, Cell type scores used to annotate the scRNA-seq dataset in **b**. For the complete list of genes used for each cell type, see Supplementary Table 1.

**Extended Data Fig. 1:**
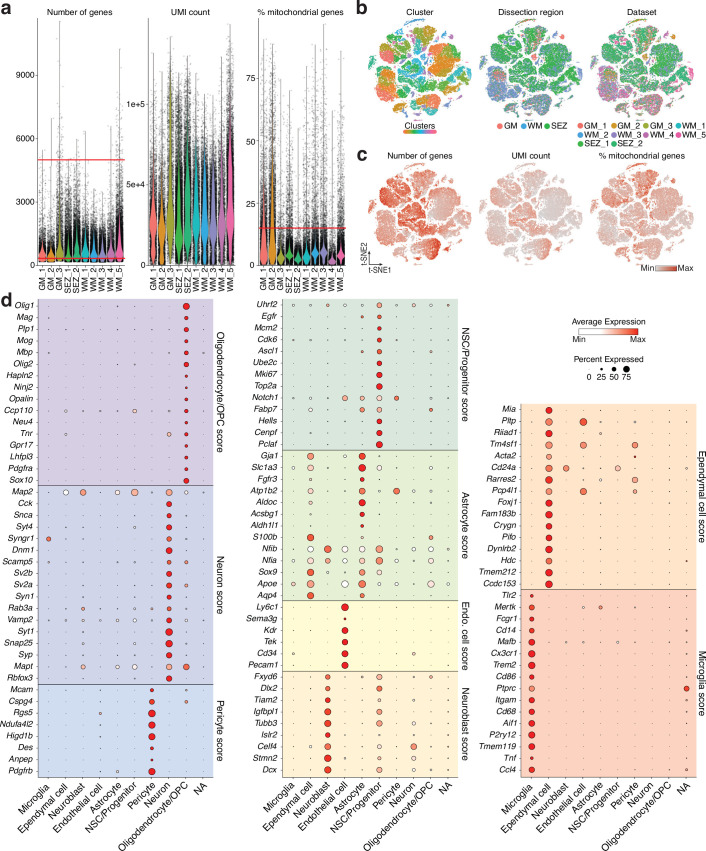
Quality control parameters of scRNA-seq samples and marker gene expression of the clusters. **a**, Violin plots showing the number of detected genes (left), UMI counts (middle), and mitochondrial gene percentages (right) for each sample. Red lines indicate thresholds: >350 and <5000 for number of genes, and <15% for mitochondrial genes. **b**, *t*-SNE visualization of selected cells, color-coded by clusters (left), dissected region (middle), and dataset (right). **c**, *t*-SNE visualization showing detected genes (left), UMI counts (middle), and mitochondrial gene percentages (right). **d**, Expression of genes used for cell type scores. Nine cell types were identified in gray matter, white matter, and subependymal zone.

To investigate differences between WM and GM astrocytes, we used an unbiased approach without antigen purification. Cells were dissociated from the WM/CC, cortical GM and subependymal zone (SEZ) of adult C57BL/6J mice (Fig. 1a). The SEZ was included because the WM may contain cells that migrated from the SEZ^19^. Quality control filtered cells based on gene counts (>350 and <5,000) and mitochondrial gene percentages (<15%; Extended Data Fig. 1a–c), with CellBender removing background noise (for example, ambient RNA)^20^. This yielded a dataset of 66,455 cells and 23,604 genes (Fig. 1b). Clustering based on the top 30 principal components (PCs) from 2,000 variable genes identified nine distinct cell types, including astrocytes and neurons, annotated using the top regulated genes and external resources (Fig. 1b). Astrocyte markers such as *Aldh1l1* and *Aldoc* were cluster-specific, while others like *Slc1a3*, *Sox9*, *Nfib*, *Nfia* and *S100b* were shared with ependymal and neural stem cells (NSCs) (Fig. 1c,d)^17,21^. Gene expression scores further validated cell type identities using established markers (Fig. 1e, Extended Data Fig. 1d and Supplementary Table 1)^10,14,22–24^.

### Region-specific and shared gene expression in astrocytes

**Extended Data Fig. 2:**
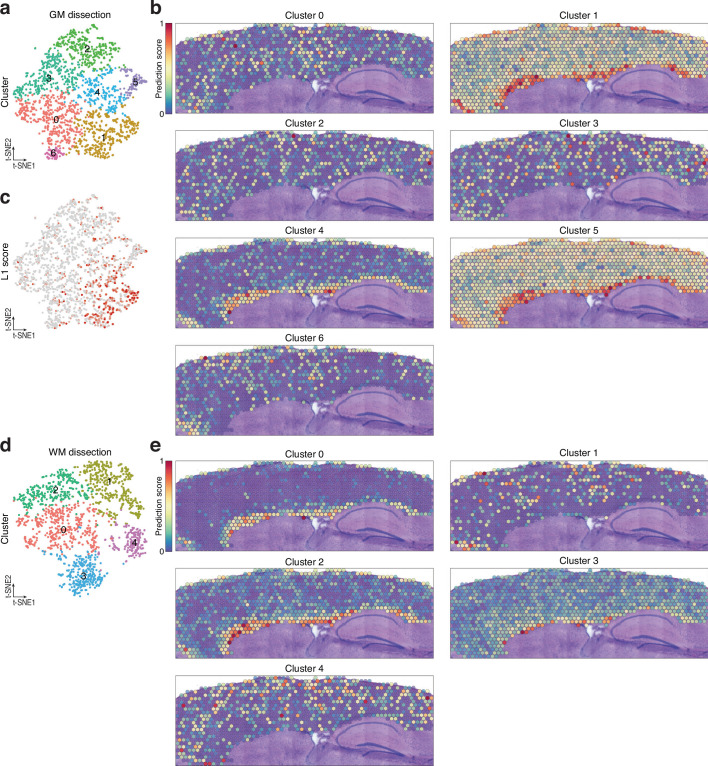
Identification of gray and white matter astrocytes using spatial transcriptomics. **a**, *t*-SNE visualization of gray matter astrocytes identified and subset from Fig. 1b. Cluster analysis revealed 7 distinct astrocyte clusters. **b**, Spatial mapping displaying the predicted position of gray matter astrocytes. **c**, *t*-SNE visualization of gray matter astrocytes showing layer 1 score. For the complete list of genes used, see Supplementary Table 1. **d**, *t*-SNE visualization of white matter astrocytes identified and subset from Fig. 1b. Cluster analysis unveiled 5 distinct astrocyte clusters. **e**, Spatial mapping illustrating the predicted position of white matter astrocytes.

To investigate astrocyte heterogeneity, we reclustered GM (1,893 cells) and WM/CC astrocytes (1,838 cells) using 25 PC dimensions of 2,000 variable genes. Spatial transcriptomics from a sagittal section of the C57BL/6J mouse brain was used to localize astrocyte clusters (Extended Data Fig. 2a–e).

Mapping the seven GM clusters revealed distinct distributions. Clusters 0, 2, 3 and 6 exhibited a scattered, salt-and-pepper pattern throughout the cortical GM, while clusters 1 and 5 showed dense labeling in the GM, with even stronger signals in the WM and layer 1 (Extended Data Fig. 2b). WM and layer 1 astrocytes have a similar molecular identity^9^. To distinguish the layer 1 signature, gene expression scores were calculated, showing higher enrichment in cluster 1 compared to cluster 5 (Extended Data Fig. 2c)^25^. Cluster 4 displayed a scattered GM distribution with the strongest signal in the WM (Extended Data Fig. 2b). These results suggest that some GM astrocytes share gene expression with WM and layer 1 astrocytes, which is consistent with previous findings^17^, although minor cross-contamination due to the proximity of the two regions cannot be excluded.

Next, we analyzed WM/CC astrocytes and identified five clusters (Extended Data Fig. 2d). Cluster 0 showed region-specific expression in the WM with some signal in layer 1 and minimal GM expression (Extended Data Fig. 2e). Clusters 2 and 3 were primarily in the WM, but also mapped to the GM, while clusters 1 and 4 exhibited a scattered distribution across the WM and GM, indicating shared gene expression or contamination from deep GM layers (Extended Data Fig. 2e). To explore this, we compared the molecular identities of GM cluster 4 with WM clusters 2 and 3 because they showed similar spatial mapping despite their different origin. Despite shared molecular features, the analysis confirmed distinction as they clustered apart, reflecting their dissection regions.

**Fig. 2:**
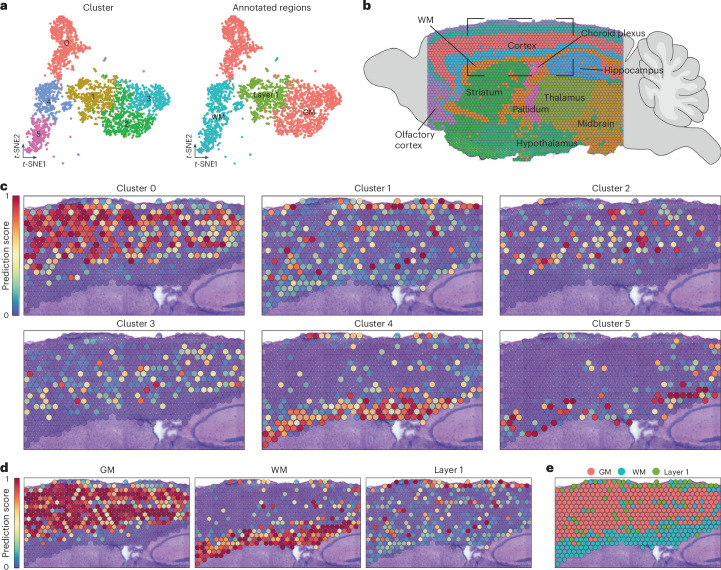
Spatial distribution of different astrocyte clusters. **a**, *t*-SNE visualization of layer 1, GM and WM astrocytes merged (from Extended Data Fig. 2) and classified into six different clusters (left). Right, *t*-SNE visualization of the final classification. **b**, Annotation of the sagittal section used for spatial mapping depicting the main regions of the mouse brain. **c**, Spatial mapping of the six astrocyte clusters identified in **a** (*t*-SNE shown on the left), illustrating their predicted localization. **d**, Spatial mapping based on the regional annotations identified in **a** (*t*-SNE shown on the right). **e**, Predicted positioning of the regional annotations identified in **a** (*t*-SNE shown on the right).

Combining GM and WM/CC astrocytes identified six clusters (Fig. 2a). Clusters 0, 2 and 3 mapped to the GM, with expression in the upper, middle and lower cortical layers, respectively (Fig. 2b,c). Clusters 4 and 5 were enriched in the WM, with cluster 4 having a broader distribution and cluster 5 being more confined (Fig. 2c). Cluster 1 showed widespread scattering with some enrichment in layer 1. These results reveal that astrocytes form both region-specific clusters and clusters with broader expression patterns, such as clusters 1 and 4, which share molecular features. Based on their spatial distribution, we assigned each cluster to a specific brain region (Fig. 2a) and visualized their cortical mapping (Fig. 2d,e).

### WM and GM astrocytes display distinct molecular signatures

**Fig. 3:**
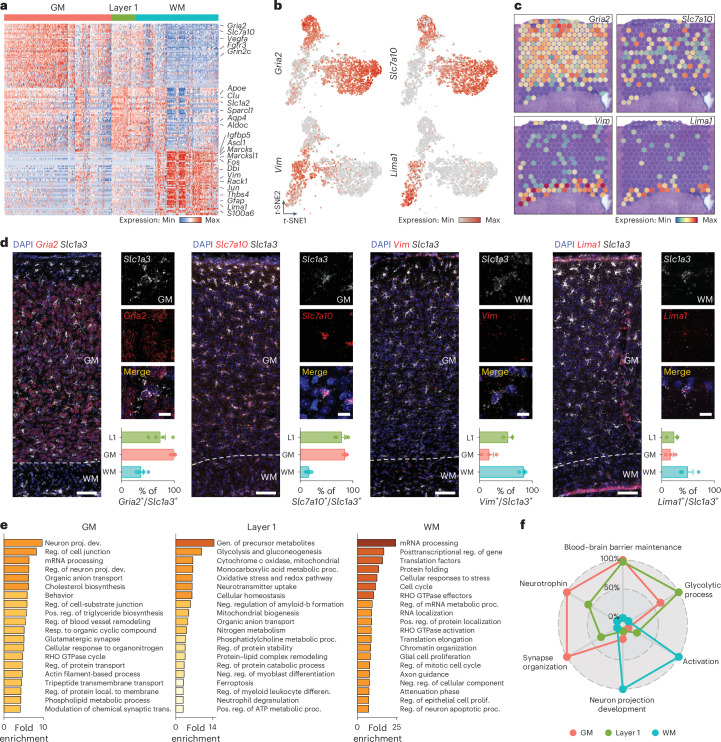
Layer 1, GM and WM astrocytes display unique molecular features. **a**, Heatmap showing the expression of 150 DEGs between layer 1, GM and WM astrocytes (*P*_val_ < 0.05, log_2_ fold change > 1). Two-sided Mann–Whitney *U*-test with Bonferroni post-hoc test. **b**, *t*-SNE visualization showing the expression levels (normalized) of two genes highly expressed in GM (*Gria2* and *Slc7a10*, top) and in WM astrocytes (*Vim* and *Lima1*, bottom). **c**, Expression levels of the same genes shown in **b** on the spatial dataset showing their expression (*Gria2* and *Slc7a10* in the GM, and *Vim* and *Lima1* in the WM). **d**, RNAscope in situ hybridization of *Gria2*, *Slc7a10*, *Vim* and *Lima1*. These results show enriched expression of *Gria2* and *Slc7a10* in GM astrocytes and enriched expression of *Vim* and *Lima1* in WM astrocytes. *n* = 4 animals (for *Vim* staining in layer 1 *n* = 2). Scale bars, 400 μm(overview) and 10 μm (insets). **e**, GO term (biological process) analysis using genes enriched in WM and GM astrocytes. **f**, Radar plot comparing gene scores for six chosen functions across layer 1, GM and WM astrocytes. The genes used to calculate these scores were obtained from six corresponding GO terms. The graphs display the mean ± s.e.m. Source data

To identify molecular differences between WM and GM astrocytes, we performed differential gene expression analysis, comparing clusters either unbiasedly (Fig. 2a left and Supplementary Table 2) or by spatial location (Fig. 2a right, Fig. 3a and Supplementary Table 3). Over 2,000 genes were differentially expressed in WM clusters 4 and 5 versus GM clusters. Focusing on the top regulated genes, we identified those enriched in WM/CC astrocytes, GM astrocytes and layer 1 (*P*_val_ < 0.05, log_2_ fold change > 1; Fig. 3a and Supplementary Table 3). WM/CC astrocyte-enriched genes included *Vim*, *Gfap*^15^, *Igfbp5*, *Dbi* and *Lima1*, while GM astrocytes exhibited elevated expression of *Gria2*, *Slc7a10*, *Fgfr3* and *Vegfa* (Fig. 3a and Supplementary Table 3). This region-specific gene expression was evident at the single-cell and spatial levels (Fig. 3b,c). RNAscope analysis showed enriched *Gria2* and *Slc7a10* expression in *Slc1a3*^+^ GM astrocytes, while *Vim* and *Lima1* were more expressed in *Slc1a3*^+^ WM/CC astrocytes (82% of all WM/CC astrocytes expressed *Vim*; Fig. 3d).

**Extended Data Fig. 3:**
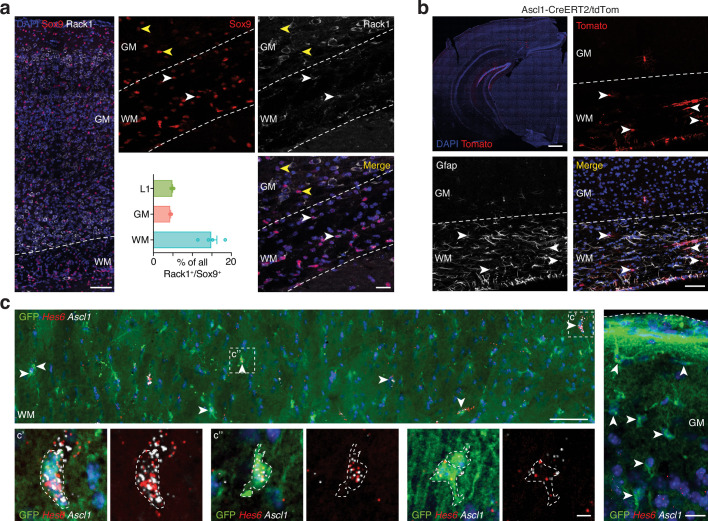
Molecular differences between layer 1, gray, and white matter astrocytes. **a**, Confocal images of sections immunostained for Sox9 and Rack1. Scale bars: 400 μm (overview), 50 μm (inset). Quantifications of Rack^+^/Sox9^+^ astrocytes in layer 1, white and gray matter. n = 4 animals. Graph display mean ± s.e.m. **b**, Confocal images from sections of Ascl1-CreERT2/tdTom mice immunostained for Gfap showing white matter astrocytes positive for both Gfap and tdTomato. Scale bars: 200 μm (overview), 50 μm (inset). **c**, RNAscope in situ hybridization of *Ascl1* and *Hes6* showing positive white matter astrocytes (on the left) and negative gray matter astrocytes (on the right) in Aldh1l1-eGFP mice. Scale bars: 50 μm (overview), 10 μm (inset). Source data

We examined gene expression differences underlying known and potential unknown functions of WM and GM astrocytes. Morphological differences were supported by high expression of cytoskeletal regulatory genes in WM/CC astrocytes (Fig. 3a). *Marcks* and *Marcksl1* mediate actin cytoskeleton crosslinking, while *Lima1* links the cadherin–catenin complex to the cytoskeleton (Fig. 3a)^26^. *Rack1*, also enriched in WM/CC astrocytes, regulates cell contacts, possibly at the nodes of Ranvier (Fig. 3a and Extended Data Fig. 3a). Additionally, *Lima1* is involved in cholesterol uptake^27^, possibly supporting WM metabolism and myelination. Gene Ontology (GO) analysis revealed metabolism-related terms enriched in WM/CC astrocytes, while GM astrocytes were associated with synaptic functions (Fig. 3e and Supplementary Table 4), emphasizing metabolic specialization in WM and predominant synaptic roles in GM astrocytes^28^. Gene expression scores based on functional genes (Supplementary Table 5) highlighted distinct profiles for astrocyte subtypes, including synapse organization, glycolytic processes and blood–brain barrier maintenance (Fig. 3f). Neuron projection development, exclusively regulated in WM/CC astrocytes, probably reflects their role at axons and the nodes of Ranvier (Fig. 3f). WM astrocytes also displayed ‘activation’ terms, similar to reactive astrocytes observed after brain injury^29^, as evidenced by higher *Gfap* and *Vim* expression.

WM/CC astrocytes showed enrichment of gliogenesis and proliferation-related terms, with genes linked to radial glial cells (RGCs), such as *Fabp7* and *Tox3*, and immediate early genes like *Fos*, *Egr1*, *Jun*, *Jund* and *Btg2* (Fig. 3a and Supplementary Tables 3 and 4), the latter having a role in neurogenesis^30^. GO terms associated with proliferation were observed in WM/CC astrocytes but not in GM astrocytes, supporting their progenitor-like state, with fate determinants including *Sox4*, *Sox11*, *Ascl1* and *Hes6* (Fig. 3a,e and Supplementary Tables 2 and 3). To confirm this, we detected Gfap^+^/tdTomato^+^ astrocytes in the WM/CC of Ascl1-CreERT2/tdTom mice, which express tamoxifen-inducible Cre recombinase under the *Ascl1* promoter 33 days after induction (Extended Data Fig. 3b). Double RNAscope analysis of *Ascl1* and *Hes6* in Aldh1l1-enhanced green fluorescent protein (eGFP) mice confirmed their coexpression predominantly in WM/CC astrocytes^17^, with no signal in the GM (Extended Data Fig. 3c). Furthermore, proliferation-specific genes like *Ccnd2* and *PCNA* were enriched in WM/CC astrocytes, suggesting plastic, RGC-like, proliferating astrocytes in the WM (Supplementary Table 3).

### Identification of a WM/CC proliferative astrocyte subtype

**Fig. 4:**
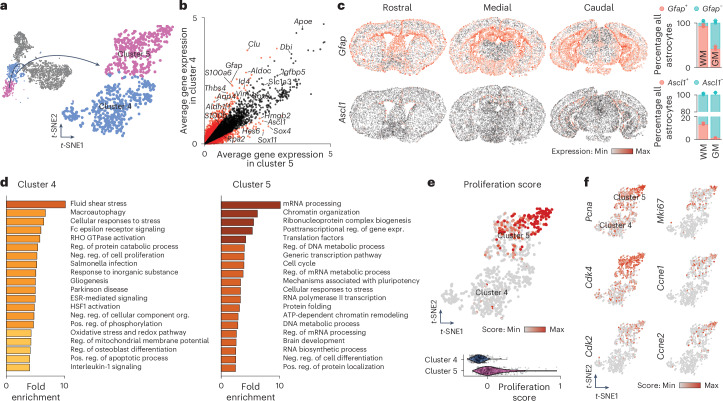
A subset of WM astrocytes exhibit proliferative capacities. **a**, *t*-SNE visualization of the two WM clusters (clusters 4 and 5). **b**, DEGs between cluster 4 and 5. The red dots represent genes that are differentially expressed between the two clusters (*P*_val_ < 0.01, log_2_ fold change > 0.35), whereas genes expressed by both WM astrocyte subtypes are represented by black dots. Two-sided Mann–Whitney *U*-test with Bonferroni post-hoc test. **c**, The Vizgen MERSCOPE single-cell spatial dataset showing expression of *Gfap* and *Ascl1* in astrocytes from three coronal sections (rostral, medial and caudal) in the WM. Right, Quantifications of the fraction of astrocytes positive for *Gfap* or *Ascl1* in GM and WM. *n* = 3 sections (one rostral, one medial and one caudal). The graphs display the mean ± s.e.m. **d**, GO term (biological process) analysis using genes enriched in clusters 4 and 5. **e**, *t*-SNE visualization and violin plots of the proliferation score in both WM clusters. For the complete gene list used for the proliferation score, see Supplementary Table 1. **f**, *t*-SNE visualization of clusters 4 and 5 showing the expression levels (normalized) of some proliferation genes used for the proliferation score.

**Extended Data Fig. 4:**
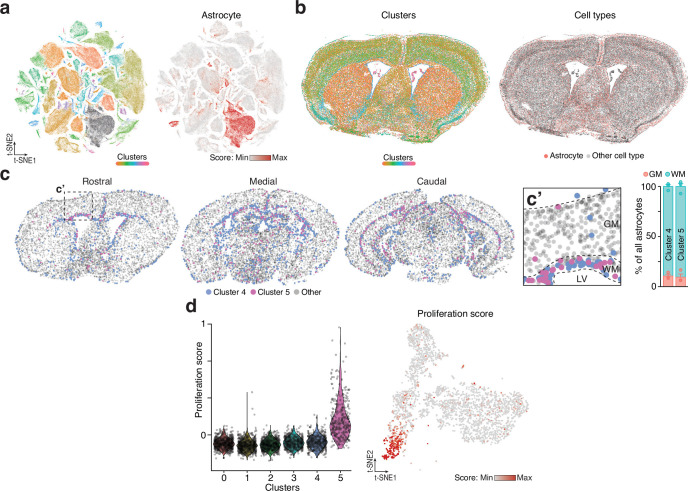
The ability to proliferate is restricted to a unique cluster of white matter astrocytes. **a**, *t*-SNE visualization of the single cell Vizgen MERSCOPE spatial dataset showing cluster analysis (on the left), and the astrocyte score (on the right) used to identify astrocytes. **b**, Spatial visualization of the clusters of the Vizgen MERSCOPE dataset (on the left) and the spatial distribution of the identified astrocytes (on the right). **c**, Predicted identity and localization of cluster 4 and 5 on the single cell Vizgen MERSCOPE spatial dataset confirming cluster 4 and 5 localization within the white matter. Graphs show the proportion of each cluster predicted in the white and gray matter. Graph display mean ± s.e.m. **d**, Violin plot (on the left) and *t*-SNE visualization (on the right) of the proliferation score in all 6 clusters of astrocytes.

To determine if high progenitor and proliferation gene expression was specific to a subset of WM/CC astrocytes, clusters 4 and 5 (Fig. 2a) were subclustered using 15 PC dimensions from 2,000 variable genes, resulting in 807 astrocytes (449 in cluster 4 and 358 in cluster 5; Fig. 4a). We analyzed differentially expressed genes (DEGs) (*P*_val_ < 0.01, log_2_ fold change > 0.35; Fig. 4b and Supplementary Table 6). Both clusters expressed typical astrocyte genes (*Vim*, *Sox9*, *Slc1a3*, *Apoe*) (Fig. 4b). Cluster 5 exhibited high expression of progenitor genes (*Sox4*, *Sox11*, *Ascl1*, *Hmgb2*), while *Gfap*, *S100a6* and *Aqp4* were enriched in cluster 4. To confirm these two clusters, we used a publicly available spatial dataset (Vizgen MERSCOPE), performing standard unsupervised clustering analysis. We identified astrocytes by calculating a score for each cell type (Extended Data Fig. 4a,b). Most *Gfap*^+^ astrocytes were primarily located in the WM (Fig. 4c). Moreover, *Ascl1*^+^ astrocytes (*Ascl1* was enriched in cluster 5) were predominantly present in the WM/CC (Fig. 4c). Predicting their position, cluster 5-like and cluster 4-like astrocytes were predominantly located in the WM/CC (Extended Data Fig. 4c,c’), confirming two distinct WM/CC astrocyte clusters and their associated gene expression using this spatial dataset with single-cell resolution.

To explore the functional implications of the two astrocyte subsets, we performed GO analysis (Fig. 4d and Supplementary Table 7). Cluster 4 exhibited GO terms enriched in macroautophagy and the regulation of catabolic processes (Fig. 4d), indicating that they may regulate myelin remodeling through autophagy. Cluster 4 also exhibited enriched GO terms for gliogenesis but no GO terms related to proliferation. The GO terms for cluster 5 are related to cell cycle and proliferation processes, including DNA metabolism and RNA processing (Fig. 4d). This was confirmed by a proliferation score based on 61 genes (Supplementary Table 1 and Fig. 4e) with increased expression of proliferation genes (Fig. 4f) in cluster 5 but not in cluster 4 or GM clusters (Extended Data Fig. 4d). These data suggest WM/CC as a further niche of proliferating astrocytes and astrogenesis.

### Cerebellar WM astrocytes have a distinct molecular signature

**Fig. 5:**
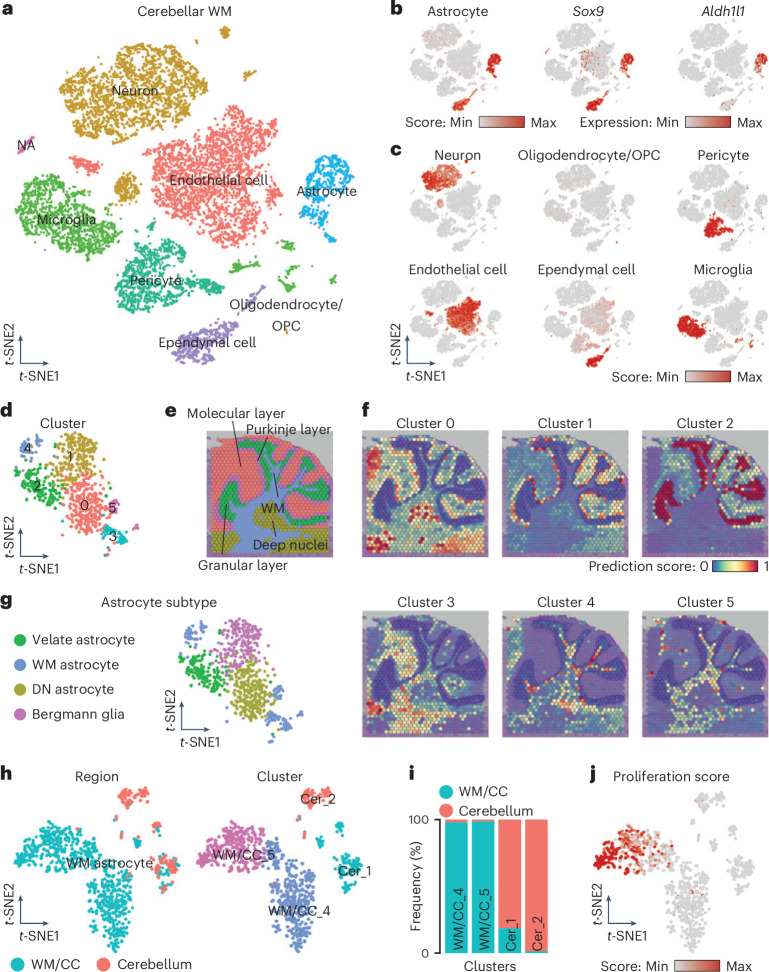
Profile of cerebellar WM astrocytes shows region-specific hallmarks. **a**, *t*-SNE visualization of scRNA-seq data from the WM of the cerebellum visualized according to cell type. **b**, *t*-SNE visualization showing the astrocyte score and expression levels (normalized) of common markers for astrocytes. **c**, Cell type scores used to annotate the scRNA-seq dataset in **a**. For the complete gene list used for each cell type, see Supplementary Table 1. **d**, *t*-SNE visualization of the astrocytes identified and subset from **a** segregated in six different clusters. **e**–**g**, Annotation of the cerebellar layers on a sagittal brain section (**e**) and spatial mapping of the six cerebellar astrocyte clusters showing their predicted localization (**f**), which was used to annotate the six different clusters (**g**). **h**, *t*-SNE visualization of the integrated dataset of WM astrocytes from the WM/CC (Fig. 4a, clusters 4 and 5) and cerebellum (**g**), showing the region of origin (left) and the cluster analysis (right). **i**, Proportion of WM astrocytes in the clusters originating from the two regions (WM/CC versus cerebellum). **j**, *t*-SNE visualization of the integrated dataset showing the proliferation score.

**Extended Data Fig. 5:**
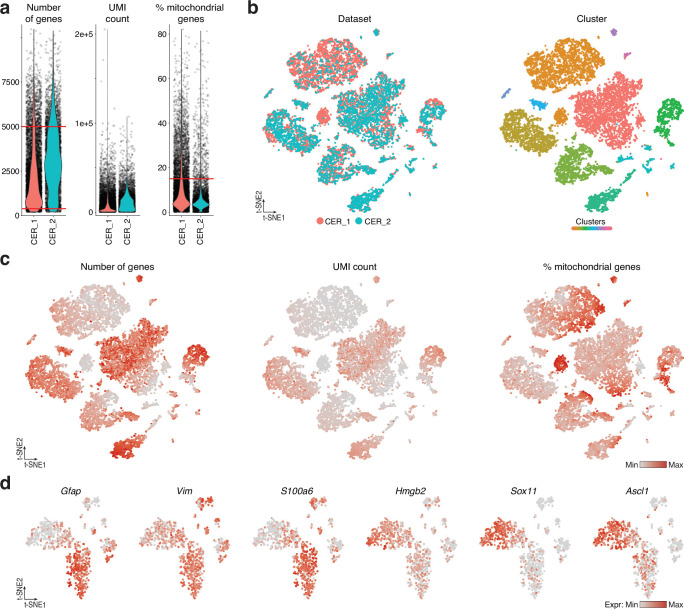
Quality assessment for the cerebellar scRNA-seq datasets and gene expression of candidate genes. **a**, Violin plots for number of detected genes (left), number of UMI counts (middle), and percentages of mitochondrial genes detected (right) for both scRNA-seq samples. Red lines indicate the thresholds set at > 350 and < 5000 for the number of genes, and < 15 for the percentage of mitochondrial genes. **b**, *t*-SNE visualization of cerebellar scRNA-seq data including all selected cells, with cells color-coded based on the dataset (left), and cluster (right). **c**, *t*-SNE visualization showing the levels of number of detected genes (left), number of UMI counts (middle), and percentages of mitochondrial genes detected (right). **d**, *t*-SNE visualization showing expression levels (normalized) of some genes highly expressed by white matter/corpus callosum astrocytes.

To determine if the described hallmarks apply to all WM astrocytes, we examined cerebellar WM using scRNA-seq. Cells were filtered using gene counts (>350 and <5,000) and mitochondrial gene percentages (<15%; Extended Data Fig. 5a–c). Two datasets from six mice each were analyzed using 30 PC dimensions of 2,000 variable genes (Fig. 5a,b). Clusters were annotated based on the top regulated genes (Fig. 5a–c). While we acknowledge that sample size may influence statistical power, we identified 810 astrocytes among 19,844 cells (Fig. 5a,b). The large neuronal cluster indicates a considerable amount of GM in our dissection; this was expected considering the slender nature of the lobular WM which posed significant challenges for dissection (Fig. 5c). Next, astrocytes were reclustered (Fig. 5d). Subsequently, we used a publicly available spatial transcriptomic dataset (‘Data availability’ and Fig. 5e,f) and found clusters 3, 4 and 5 mainly mapping to the cerebellar WM, with cluster 4 showing additional signal in the deep cerebellar nuclei (Fig. 5f). Clusters 1 and 2 were primarily associated with cerebellar cortical layers, like the molecular or granular layer or with the deep cerebellar nuclei (cluster 0), confirming some degree of contamination from extra WM territories (Fig. 5f). Spatial mapping enabled us to identify different astrocyte types (for example, Bergmann glia, WM or velate astrocytes) in the cerebellum (Fig. 5g), a region where identifying astrocyte subtypes with common markers has been difficult.

Next, we merged and reclustered WM astrocytes from the WM/CC and cerebellum, identifying four distinct clusters, that is, two per region (WM/CC_4, WM/CC_5, Cer_1 and Cer_2; Fig. 5h). Only cluster Cer_1 included some WM/CC astrocytes (Fig. 5i). As in astrocytes from the WM/CC, *Vim* was also expressed in cerebellar WM astrocytes, whereas *Gfap* was less detectable (Extended Data Fig. 5d). Interestingly, we also noted the expression of some genes characteristic for NSCs, such as *S100a6* (ref. ^31^) and *Hmgb2* (Extended Data Fig. 5d). However, other genes upregulated specifically in the proliferative WM/CC cluster, like *Sox11* and *Ascl1*, were not enriched in cerebellar WM astrocytes (Extended Data Fig. 5d), which also had a low proliferation score (Fig. 5j). These findings indicate that WM/CC and WM cerebellar astrocytes have different molecular features, with some commonalities between WM/CC cluster 4 and the cerebellar clusters.

### Cross-species comparison to human WM astrocytes

**Fig. 6:**
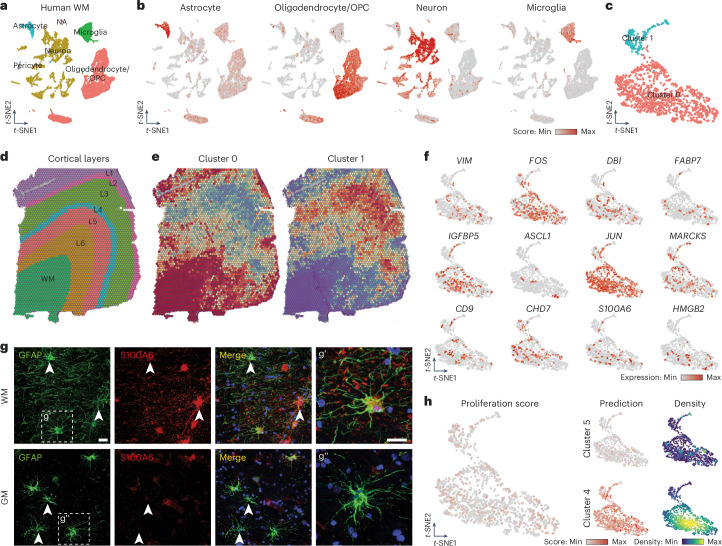
Human WM astrocytes share gene expression pattern with their mouse counterparts. **a**, *t*-SNE visualization of snRNA-seq data from the human WM visualized according to cell type. **b**, *t*-SNE visualization showing the cell type scores used to annotate the snRNA-seq dataset. For the complete gene list used for each cell type, see Supplementary Table 1. **c**, *t*-SNE visualization of astrocytes identified and subset from **a** segregated into two different clusters. **d**, Annotation of a human section showing the localization of the WM and the different GM cortical layers. **e**, Spatial mapping of the two human astrocytes clusters (0 and 1) showing their predicted localization. **f**, *t*-SNE visualization showing expression levels (normalized) of some genes highly expressed by WM astrocytes identified in the mouse dataset. **g**, Confocal images of human WM and GM double-stained for S100A6 and GFAP. Scale bars, 50 μm (overview) and 20 μm (insets). **h**, *t*-SNE visualization showing the proliferation score (left) and the prediction score with density distribution (right) for murine clusters 4 and 5 of WM astrocytes. The density distribution plot highlights areas of higher and lower concentration of cells displaying high and low levels of prediction scores in the dataset.

**Extended Data Fig. 6:**
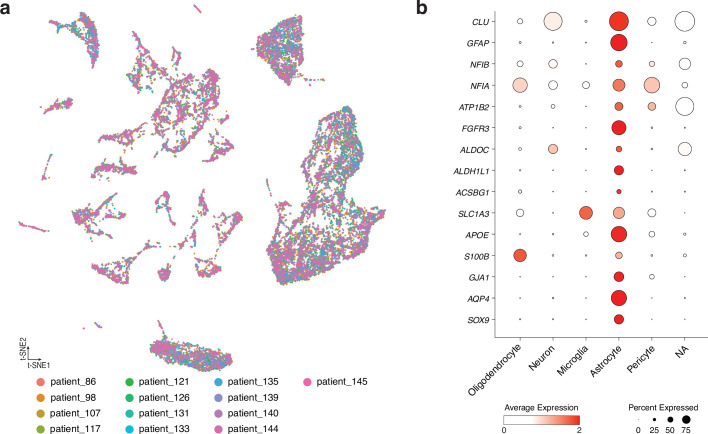
The human dataset. **a**, *t*-SNE visualization of snRNA-seq data from the human white matter, visualized by patient origin. Cells were collected from 13 different control patients. **b**, Cell type-specific expression of genes used to calculate the astrocyte score.

To investigate whether WM/CC astrocyte subtypes are present in human WM, we analyzed a single-nucleus RNA sequencing (snRNA-seq) dataset from the WM of 13 controls from a cohort with multiple sclerosis (Roche dataset, EGAD00001009169). The dataset included 27,300 nuclei and 24,809 detected genes (Fig. 6a and Extended Data Fig. 6a). Using 30 PC dimensions of 3,000 variable genes, we identified clusters based on marker expression (Fig. 6a,b and Extended Data Fig. 6b). Subsetting and reclustering astrocytes with 25 PC dimensions revealed two main clusters: cluster 0 (WM) and cluster 1 (GM), as confirmed by mapping single-nucleus expression data to a human spatial gene expression dataset (Fig. 6c–e)^32^.

To explore shared and species-specific traits, we cross-referenced mouse WM/CC clusters 4 and 5 with human WM cluster 0, examining highly expressed genes in the human context. In human WM astrocytes, these genes exhibited a scattered ‘salt-and-pepper’ expression pattern. Immediate early genes like *FOS* and progenitor genes such as *S100A6* and *HMGB2* were also expressed (Fig. 6f). Staining human brain sections for S100A6 and glial fibrillary acidic protein (GFAP) confirmed their coexpression by WM but not GM astrocytes (Fig. 6g). However, the proliferation score in the human dataset revealed minimal expression of these genes (Fig. 6h, left). We subsequently used the molecular signature of murine clusters 4 and 5 to predict the identity of human WM astrocytes, finding them more cluster 4-like (Fig. 6h, right). Thus, we found a shared set of WM astrocytes present across species (cluster 4) and a murine-specific subset with a pronounced proliferation score (Fig. 4e).

### WM/CC astrocytes proliferate in vivo

**Fig. 7:**
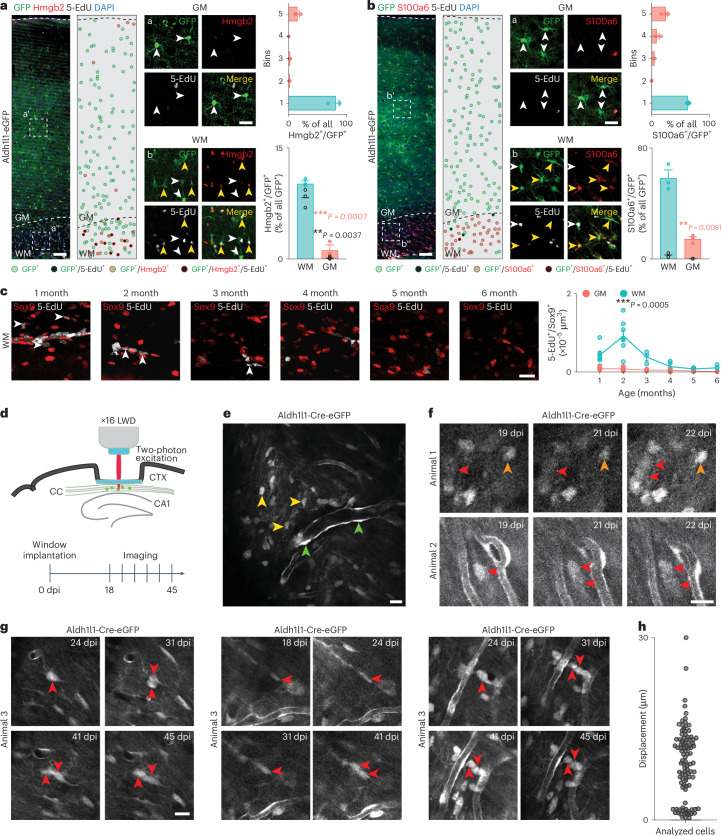
WM/CC astrocytes proliferate in vivo*.* **a**,**b**, Confocal images of Aldh1l1-eGFP mice immunostained for Hmgb2 (**a**) and S100a6 (**b**) after 4 weeks of 5-EdU administration. The dots represent astrocytes (see the color code below the image). Quantifications showed Hmgb2^+^/GFP^+^ (**a**) or S100a6^+^/GFP^+^ (**b**) astrocytes in a column (top) and proportions in the WM/GM (bottom). The black dots represent the amount of Hmgb2^+^/5-EdU^+^/GFP^+^ (**a**) and S100a6^+^/5-EdU^+^/GFP^+^ (**b**) astrocytes. *n* = 3 animals. Two-sided Student’s *t*-test. Scale bars, 100 μm and 50 μm (insets), and 200 μm (overview), 50 μm (inset). **c**, Confocal images of Sox9^+^/5-EdU^+^ astrocytes in the WM of C57BL/6J mice at different ages (1–6 months) and their quantification (performed after 24 h 5-EdU administration and a subsequent 24 h chase). *n* ≥ 3 animals (for the details, see ‘Statistics and reproducibility’ in the Methods). Two-way analysis of variance (ANOVA) with Šídák’s post-hoc test. Scale bar, 20 μm. **d**, Schematic representation of the window implantation and workflow used for the chronic live imaging. **e**, Overview image of an exemplary field of view showing the vascular pattern, which was used as a landmark in repeated imaging sessions. Parenchymal (yellow arrowhead) and juxtavascular (green arrowhead) astrocytes are indicated. **f**,**g**, Two-photon images of Aldh1l1-Cre-eGFP mice WM astrocytes at different days after implantation. The red arrows indicate GFP^+^ astrocytes that underwent division. The orange arrow indicates a nondividing GFP^+^ astrocyte. *n* = 5 animals were analyzed. Scale bar, 20 μm. **h**, Migration of astrocytes between 18 and 31 days after implantation in µm. *n* = 100 cells. Graphs display the mean ± s.e.m. *P ≤ 0.05, **P ≤ 0.01, ***P ≤ 0.001. dpi, days after (post) implantation. Source data

**Extended Data Fig. 7:**
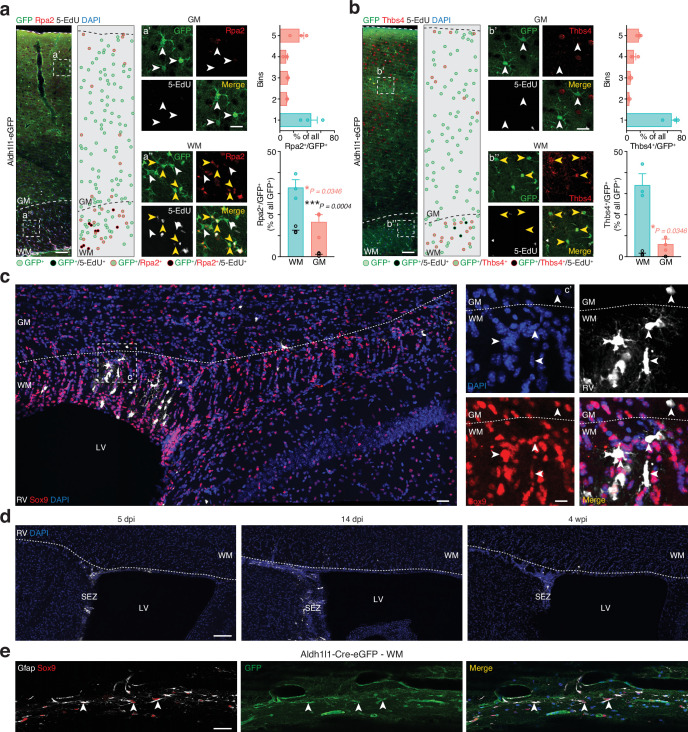
Characterization of proliferative white matter astrocytes. **a**,**b**, Confocal images of Aldh1l1-eGFP mice immunostained for Rpa2 (**a**) and Thbs4 (**b**) after 4 weeks of 5-EdU administration. Dots represent astrocytes in the images (see color code below the image). Scale bars: 100 μm and 50 μm (insets). Quantifications show Rpa2^+^/GFP^+^ (**a**) or Thbs4^+^/GFP^+^ (**b**) astrocytes in a column (top graphs) and proportions in white/gray matter (bottom graphs). Black dots indicate the amount of Rpa2^+^/5-EdU^+^/GFP^+^ (**a**) Thbs4^+^/5-EdU^+^/GFP^+^ (**b**) astrocytes. n = 3 animals. Two-sided Student’s t test. Scale bars: 200 μm (overview), 50 μm (inset). **c**, Confocal images of Sox9 and RFP from mice 14 days after retrovirus (RV) injection into the white matter to label dividing cells. Overview of the ventricular zone/white matter (on the left) and zoom (on the right). Scale bars: 50 μm and 10 μm (insets). **d**, Confocal images of mice 5 days, 14 days, and 4 weeks after RV injection into the SEZ. Scale bars: 100 μm. **e**, Confocal images of Aldh1l1-Cre-eGFP white matter stained for Sox9, Gfap, and GFP. Scale bars: 50 μm. Graphs display mean ± s.e.m. * p ≤ 0.05; ** p ≤ 0.01; *** p ≤ 0.001. Source data

To determine if cluster 5 WM/CC astrocytes proliferate, we provided the thymidine analog 5-ethynyl-2′-deoxyuridine (5-EdU) to Aldh1l1-eGFP mice for 4 weeks in drinking water^17^, followed by immunostaining for markers identified for cluster 5 (that is, *Hmgb2* and *Rpa2*) or cluster 4 (that is, *S100a6* and *Thbs4*) WM/CC astrocytes (Fig. 7a,b and Extended Data Fig. 7a,b). GFP^+^ astrocytes expressing these four markers were mainly located in the WM/CC, with only a minority observed in the superficial layer of the GM cortex (Fig. 7a,b and Extended Data Fig. 7a,b). Most GFP^+^/Hmgb2^+^ or GFP^+^/Rpa2^+^ astrocytes were also positive for 5-EdU (Fig. 7a and Extended Data Fig. 7a). In contrast, GFP^+^/S100a6^+^ or GFP^+^/Thbs4^+^ astrocytes were rarely labeled by 5-EdU (Fig. 7b and Extended Data Fig. 7b). We also injected murine leukemia virus MLV-based retrovirus (RV) expressing red fluorescent protein (RFP), which incorporates its retrotranscribed genome only in dividing cells^33^, labeling dividing cells and their progeny. After 14 days of injection, many RFP-expressing cells were positive for Sox9, confirming astrocyte identity (Extended Data Fig. 7c). We also found RFP^+^ oligodendrocyte progenitors (OPCs) and doublecortin-expressing neuroblasts, but these were not Sox9^+^. Together, three independent methods—gene expression, 5-EdU and RV incorporation—showed astrocytes from cluster 5 proliferating. To understand if those astrocytes originated from the nearby SEZ, we injected the same RV into the SEZ and analyzed three time points: 5 days, 14 days and 4 weeks after injection. We observed labeled cells in the SEZ, along the rostral migratory stream and in the olfactory bulb, confirming successful labeling. At no time point, did we find RFP^+^ astrocytes in the WM/CC (Extended Data Fig. 7d).

**Extended Data Fig. 8:**
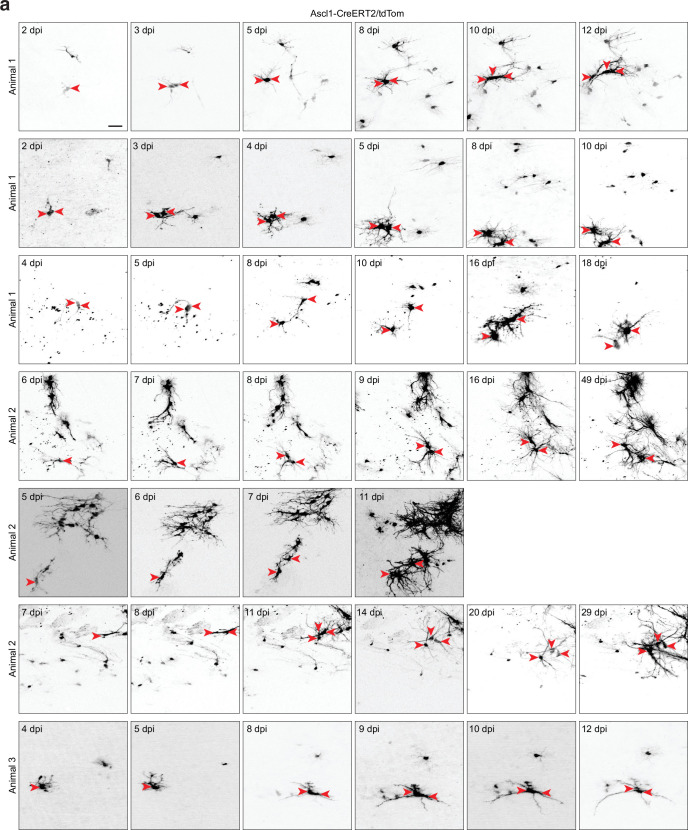
Live imaging of white matter astrocytes using Ascl1-CreERT2/tdTom mice. **a**, White matter astrocytes are detectable after recombination in Ascl1-CreERT2/tdTom mice. Recombination is induced by two injections of tamoxifen at 2 and 4 days after implantation of the window. In vivo 2-photon imaging starts earliest at 2 days and is carried out up to 49 days. Sequences of field views in the white matter indicate the proliferation of astrocytes (red arrowheads). The imaging time point, counted after the first tamoxifen injection, is displayed (dpi). Cells acquire astrocytic morphology over time. Note that in some examples, two closely associated cells seen in the first timeframe move apart and further develop more complex morphologies, indicative of two daughter cells after division. Examples from three different animals are displayed. Single images are collapsed z-stacks of in vivo imaging data. Scale bar: 20 μm.

To determine the dynamics of proliferating astrocytes with age, mice of different ages were given 5-EdU in drinking water for 24 h and euthanized 24 h later. Even after this short 5-EdU pulse, some Sox9^+^ astrocytes were 5-EdU^+^, indicating that astrocytes proliferate rather quickly (Fig. 7c). Most fast-proliferating astrocytes were observed in the WM/CC at 2 months of age, slowly declining later with a very low level after 4 months (Fig. 7c). Astrocytes from the GM proliferated rarely during the analyzed period (Fig. 7c). Compelling evidence for cell proliferation was obtained using live imaging. As the WM is deeply embedded in the brain, we removed the overlying cortical GM (Fig. 7d) as described previously^34^ and imaged GFP^+^ cells in Aldh1l1-Cre-eGFP mice over several days (Fig. 7e). Staining brain sections with Sox9 and Gfap confirmed that GFP^+^ cells in the WM were astrocytes (Extended Data Fig. 7e). Interestingly, we observed several examples of a single astrocyte dividing into two daughter cells, both within a relatively short observation period of 4 days (Fig. 7f) and during long observation periods of 21 days (Fig. 7g), demonstrating astrocyte proliferation in the WM/CC. Analyzing the lateral dispersion of astrocytes revealed only short-distance movements up to 30 μm (Fig. 7h). Additionally, live imaging of Ascl1-CreERT2/tdTom mice after tamoxifen induction revealed proliferating astrocytes in the WM/CC in vivo, confirming their origin from Ascl1^+^ cells, that is, from cluster 5 (Extended Data Fig. 8a)^35^. Thus, astrocytes proliferate only in specific regions of the adult brain, interestingly all in the forebrain.

### Progeny of proliferating WM/CC astrocytes

**Fig. 8:**
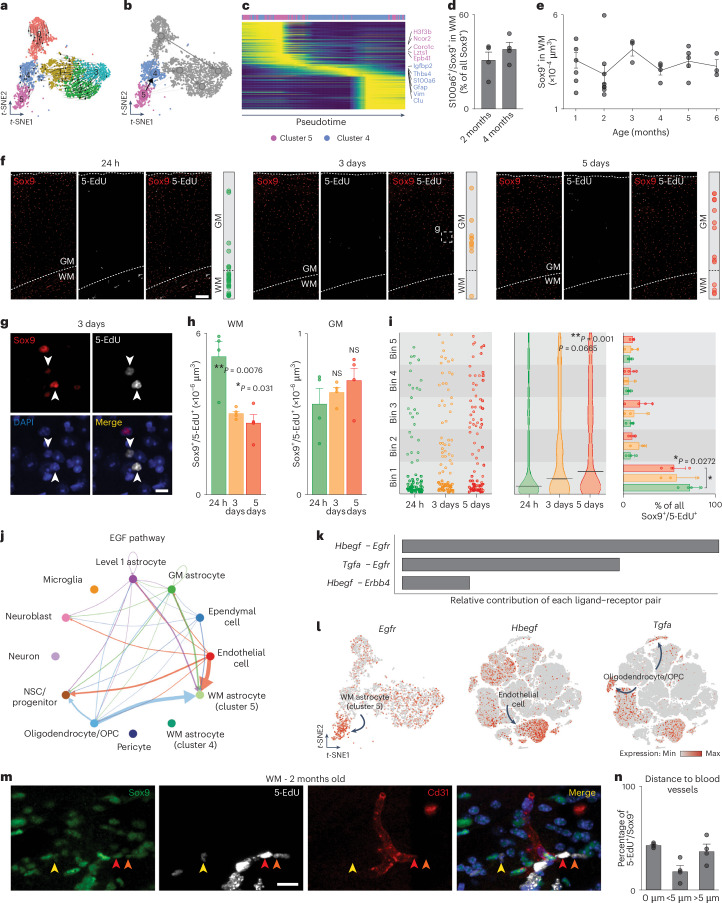
Progeny of WM astrocytes and regulatory signals controlling their proliferation. **a**, *t*-SNE plot of the isolated astrocytes showing RNA velocities. **b**, PAGA analysis showing differentiation from cluster 5 to cluster 4. **c**, Pseudotime heatmap of the top 3,000 most variable genes along the differentiation axis. **d**, Analysis of S100a6^+^/Sox9^+^ astrocytes in the WM at 2 and 4 months. *n* = 4 animals. **e**, Quantifications of Sox9^+^ astrocytes over time in the WM. *n* ≥ 3 animals (for details, see ‘Statistics and reproducibility’ in the Methods). One-way ANOVA with Tukey post-hoc test. **f**, Confocal images immunostained for Sox9. 5-EdU was administered in drinking water for 24 h and staining was performed 24 h, 3 days and 5 days after 5-EdU administration. Scale bar, 100 μm. **g**, Closer view of Sox9^+^/5-EdU^+^ astrocytes in the GM after 3 days. Scale bar, 20 μm. **h**, Analysis of Sox9^+^/5-EdU^+^ astrocytes over time. *n* = 4 animals. One-way ANOVA with Tukey post-hoc test. **i**, Position of Sox9^+^/5-EdU^+^ astrocytes over time from the WM (bin 1) to the superficial layer of the cortex (bin 5). *n* = 3 animals. One-way (middle) and two-way (right) ANOVA with Tukey post-hoc test. **j**, Cell–cell communication mediated by the epidermal growth factor (EGF) pathway, with the arrow thickness indicating strength. **k**, Contribution of ligand–receptor pair in EGF signaling. **l**, *t*-SNE plots showing *Egfr*, *Hbegf* and *Tgfa* expression in astrocytes (left) and all cells (middle and right). **m**, Confocal images of WM immunostained for Sox9,5-EdU and Cd31. Scale bar, 20 μm. **n**, Analysis of Sox9^+^/5-EdU^+^ astrocytes in contact (0 µm), closer (<5 µm) and far away (>5 µm) from blood vessels (*n* = 4 animals). The graphs show the mean ± s.e.m. *P ≤ 0.05, **P ≤ 0.01, ***P ≤ 0.001. Source data

To identify the progeny of WM astrocytes, we performed RNA velocity analysis comparing unspliced and spliced mRNAs levels. Interestingly, the arrows point from cluster 5 toward clusters 4 but also toward GM clusters (Fig. 8a). Based on RNA velocity, we calculated connectivity using partition-based graph abstraction (PAGA) and inferred the direction determining possible differentiation trajectories. Cluster 5 mainly connected to cluster 4, but it also linked to clusters 0, 1 and 2 via cluster 4 (Fig. 8b). We further identified a set of genes with pronounced dynamic behavior in this transition that may underlie this differentiation process (Fig. 8c and Supplementary Table 8). For example, genes like *S100a6*, *Gfap* and *Vim* are upregulated along pseudotime during this process, suggesting that proliferating astrocytes of cluster 5 give rise to cluster 4 astrocytes. To test this hypothesis, we quantified S100a6^+^ astrocytes (cluster 4 marker) at the proliferation peak (2 months) and at 4 months, when proliferation is greatly reduced. We observed a slight increase in S100a6^+^ astrocytes at the later time point, indicating possible generation of cluster 4 astrocytes, potentially overlaid with some progeny dying or migrating away (Fig. 8d). However, this increase did not affect the total number of astrocytes in the WM/CC, which were constant over time (Fig. 8e).

**Extended Data Fig. 9:**
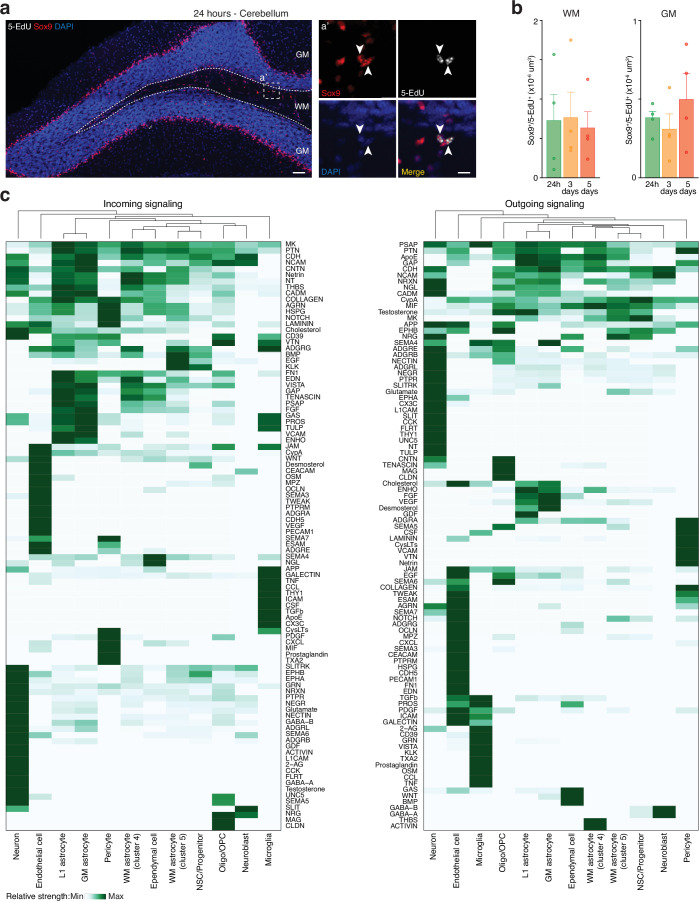
White matter astrocytes from the cerebellum do not proliferate. **a**, Confocal images of cerebellar sections immunostained for Sox9 and 5-EdU after 5-EdU administration in drinking water for 24 h. Staining was performed at 24 h, 3 days, and 5 days. Scale bars: 100 μm and 10 μm (insets). **b**, Quantifications of Sox9^+^/5-EdU^+^ astrocytes in cerebellar white and gray matter over time. n = 4 animals. One-way ANOVA with Tukey post-hoc test. Graphs display mean ± s.e.m. **c**, Heatmaps of signaling pathways showing relative strength of incoming (left) and outgoing (right) signals. Source data

To trace the progeny of cluster 5, we performed a birth dating experiment by administrating 5-EdU in drinking water for 24 h followed by a 24 h, 3-day and 5-day chase (Fig. 8f,g). The number of Sox9^+^/5-EdU^+^ astrocytes in the WM/CC significantly decreased over time, while the number in the GM increased, although not significantly (Fig. 8h). When analyzing the position of Sox9^+^/5-EdU^+^ astrocytes overtime, 5-EdU^+^ labeled astrocytes tended to enter the GM on day 3 and a greater proportion was found toward the more superficial layers of the cortex by day 5 (Fig. 8i). This observation aligns with the PAGA analysis, showing that some WM/CC astrocytes differentiate into GM astrocytes. As our analysis suggested that cerebellar WM astrocytes were less proliferative, we performed the same 5-EdU pulse-chase experiment. Only very few Sox9^+^/5-EdU^+^ astrocytes were detected in the cerebellar WM (Extended Data Fig. 9a); the few labeled cells showed no changes in position from WM to GM (Extended Data Fig. 9b). These data propose the interesting concept that only forebrain astrocytes proliferate and give rise to astrocytes, some of which may translocate to the GM. Notably, such migration was not observed using live imaging; however, it may have been compromised by removal of most of the GM.

### Signals regulating WM/CC astrocyte proliferation

To explore, if a specific cell type in the WM communicates with proliferating astrocytes, we performed a CellChat analysis identifying intercellular communication networks. Among the incoming signals, Bmp, Klk and Egf signaling were specific for proliferating cluster 5 astrocytes (Extended Data Fig. 9c). A detailed analysis of the EGF pathway revealed that the incoming signal mainly originated from oligodendrocytes/OPCs and endothelial cells (Fig. 8j). The ligand–receptor interaction identified Egfr as the receptor and Hbegf and Tgfa as ligands (Fig. 8k). Cluster 5 astrocytes expressed *Egfr*, whereas endothelial cells and oligodendrocytes/OPCs expressed the corresponding ligands (Fig. 8l). Therefore, we investigated the proximity of proliferating astrocytes (Sox9^+^/5-EdU^+^) to blood vessels (stained for Cd31) and found that 62% were located on or close (<5-µm distance) to blood vessels. These data are in line with the concept that proliferating astrocytes in the WM/CC might receive signals to enter the cell cycle from endothelial cells (Fig. 8m,n).

## Discussion

To explore WM astrocytes, we adopted an unbiased approach sampling all astrocyte subsets using a dissociation protocol preserving vulnerable cell types in the densely packed WM. Using scRNA-seq and spatial transcriptomics across WM, GM and SEZ, we identified four GM and two WM/CC astrocyte subtypes. The GM subtypes exhibited layer-specific localization, in agreement with previous work^9,10^. Notably, some of these subtypes displayed more widespread predicted positions, which are consistent with our recently proposed concept of some astrocytes sharing molecular characteristics being more widespread, while others are more specialized^17^. This entails that certain astrocyte subtypes may perform general functions, resulting in broader expression similarities^9,17,36,37^. In contrast, other astrocytes have more regionalized expression characteristics, indicating specialized functions. This was particularly intriguing for WM/CC astrocytes, where cluster 4 displayed widespread localization throughout the forebrain, while cluster 5 was highly specific to the cortical WM/CC. Interestingly, cluster 4 was shared across species, including human forebrain WM.

A primary objective of this study was to understand the distinctive characteristics of WM astrocytes. Unlike GM astrocytes, which interact at synapses, WM astrocytes interact with neurons at the nodes of Ranvier, oligodendrocytes and vasculature, supporting network activity and myelination. GO analysis confirmed synaptic transmission in GM astrocytes, while WM/CC astrocytes specialized in maintaining neural integrity and myelination^38–40^. Compared to GM astrocytes, WM astrocytes exhibited lower levels of glutamate metabolism and ATP^28,41^. Specifically, GM astrocytes expressed more of, for example, *Atp1a2*, whereas GO terms related to ‘glycogen metabolism’, ‘glycogen breakdown’ and ‘regulation of amide metabolic process’ were significantly enriched in WM/CC astrocytes. The latter may contribute to maintaining axonal function. Additionally, we observed higher expression of genes like *Lima1*, which are involved in cholesterol metabolism^27^. Many genes involved in cytoskeleton regulation, including *Rack1*, were differentially expressed between WM and GM astrocytes, probably influencing their distinct morphology.

Our dataset offers insights into both the general differences between WM and GM astrocytes and the heterogeneity within WM astrocytes. WM/CC cluster 4 astrocytes predominantly have functions related to cell contacts (GO term: tight junction), axonal support (GO terms: axon guidance, nervous system development, neurotrophin signaling pathway) and metabolism (GO terms: amide biosynthetic process, glycogen metabolism, regulation of metal ion transport). For instance, Cd63, a lysosome-associated membrane protein involved in gliotransmitter release from astrocytes, may fulfill a general role in these processes^42^. Conversely, astrocytes from cluster 5 exhibited higher expression of immediate early genes, RGCs, progenitor fate determinants and GO terms related to cell proliferation.

WM/CC astrocyte proliferation was confirmed using 5-EdU incorporation, viral labeling and live imaging. Proliferation was restricted to astrocytes from cluster 5, marked by genes like *Hmgb2*, *Ascl1*, *Hes6* and *Rpa2*, highlighting the WM/CC as a niche for region-specific astrocyte proliferation.

Proliferation of WM/CC astrocytes probably supports postnatal CC growth, peaking in young mice and declining by 4 months^43^. This aligns with the forebrain-restricted adult astrogenesis observed in neurogenic niches like the diencephalon^17^, DG hilus^44^ and SEZ^21^. This may also explain the absence of proliferation in the human WM, given the older age of individuals studied. Indeed, our data show an age-dependent peak of fast-proliferating astrocytes gradually declining, and persisting at a low level after 4 months of age in mice. However, the slow proliferation kinetics of astrocytes may well continue into later stages as observed in the diencephalon^17^. Notably, adult neurogenesis is also restricted to the forebrain in mammals, highlighting that the forebrain is particularly capable of prolonged addition of neural cells, including oligodendrocytes, neurons and now also astrocytes. Like adult neurogenesis, newly generated astrocytes could enhance plasticity, influencing synaptic remodeling by regulating synaptic connectivity and behaviors. Astrocyte proliferation has been observed in the DG, where synaptic formation, plasticity and remodeling are crucial for the formation of new memories throughout life^45^. The proliferative capacity of WM/CC astrocytes may be crucial in situations such as after acute brain injury or during disease, similar to the roles observed for GM reactive astrocytes^46^.

In addition, our data suggest that newly generated astrocytes may migrate between regions. RNA velocity analysis revealed heightened values in WM/CC cluster 5. This transcriptional flow was directed toward cluster 4 and subsequently to other GM clusters and supported by 5-EdU pulse-chase experiments showing that some Sox9^+^/5-EdU^+^ astrocytes change position from the WM/CC into the cortical GM. Although migration could not be observed in our live imaging setup because of GM removal, future studies using three-photon microscopy would be better suited to test the prediction of the WM/CC serving as a reservoir for new astrocytes in the young adult forebrain. Interestingly, WM/CC astrocyte proliferation is regulated by endothelial cells and oligodendrocytes/OPCs through the EGF signaling pathway, highlighting the complex regulatory environment in the WM/CC that controls astrocyte proliferation and potential migration.

Notably, WM/CC astrocytes differed profoundly from those in the cerebellum, where we identified two distinct clusters. Our findings suggest region-specific WM astrocytes across different brain areas, each with distinct functional roles^47^. Cross-species comparisons showed conserved traits between murine cluster 4 and human WM astrocytes. These findings highlight evolutionary conservation in WM astrocyte functions, including cell contacts, metabolism and axonal support.

WM/CC astrocytes are less heterogeneous than GM astrocytes, with murine WM/CC astrocytes divided only into two main clusters, while human WM astrocytes from our dataset formed a single large cluster. Interestingly, a recent study found that human WM astrocytes differ mainly depending on their region of origin^47^. They identified two main WM astrocyte clusters from the neocortex, two from the cerebellum and a third small cluster shared between both regions, supporting our cross-species observation. The consistency in identifying specific astrocyte clusters in different brain regions and species underlines the evolutionary conservation and functional significance of these populations. This enhances our understanding of astrocyte diversity and their specialized roles in several CNS regions, providing a broader perspective on the functional heterogeneity of WM astrocytes.

## Methods

### Experimental animals

C57BL/6J mice (Charles River Laboratories), Aldh1l1-Cre (B6;FVB-Tg(Aldh1l1-cre)JD1884Htz/J^48^,The Jackson Laboratory)) crossed to the CAG-eGFP reporter line (FVB.B6-Tg(CAG-cat,-EGFP)1Rbns/KrnzJ, The Jackson Laboratory)^49^, Aldh1l1-eGFP mice^50^ (Tg(Aldh1l1-EGFP)OFC789Gsat/Mmucd, Gensat Project) and Ascl1-CreERT2 mice (*Ascl1*^*tm1.1*^^(*Cre/ERT2*)*Jejo*^/J^51^, The Jackson Laboratory) crossed to CAG-tdTomato (B6.Cg-*Gt*(*ROSA*)*26Sor*^*tm14(CAG-tdTomato)Hze*^/J^52^; The Jackson Laboratory) were used. Mice were 2–3 months old, except for the age-dependent and imaging experiments with C57BL/6J mice aged between 1 and 6 months. Both sexes were included unless stated otherwise. Genotypes were determined with following primers: Aldh1l1-eGFP: forward TTCACCTTGATGCCGTTCT, reverse GCCGCTACCCCGACCAC, Aldh1l1-Cre-eGFP: forward CCTGTCCCCTTGCACAGTAG, mutant reverse CGGTTATTCAACTTGCACCA, wild-type reverse GTAAACCTCCTGGCCAAACA; CAG-eGFP: forward CTGCTAACCATGTTCATGCC, reverse GGTACATTGAGCAACTGACTG. Mice were kept at the Core Facility Animal Models, Biomedical Center, Faculty of Medicine, LMU Munich under specific pathogen-free conditions and housed in groups of 2–3 animals in individually ventilated cage systems in a room maintained at a temperature of 22 ± 2 °C and 55 ± 10% relative humidity, with a 12 h:12 h light:dark cycle. Mice had free access to water and were fed standard rodent chow (Altromin, 1310M). Experimental procedures were performed in accordance with animal welfare policies and were approved by the Government of Upper Bavaria (Germany).

### Treatment and surgical procedures

#### Labeling proliferating cells

5-EdU (Thermo Fisher Scientific) was administered via drinking water at a concentration of 0.2 mg ml^−1^ containing 1% sucrose for 24 h or 4 weeks.

#### Retrovirus injection

Murine leukemia virus-based RV containing RFP (RV-CAGmScarlet) was injected into the WM/CC or SEZ of C57BL/6J mice. Mice received an intraperitoneal injection containing fentanyl (0.05 mg kg^−1^, Janssen), midazolam (5 mg kg^−1^, Roche) and medetomidine (0.5 mg kg^−1^, Fort Dodge). RV (titer: 2.0 × 10^9^ plaque forming units (PFUs) ml^−1^) injection was performed with coordinates relative to bregma using an automated nanoinjector (Nanoliter 2010, World Precisions Instruments) at a slow speed (40 nl min^−1^): anteroposterior = −1.0; mediolateral = ± 0.8; dorsoventral = −1.5 (WM/CC) and anteroposterior = +0.6; mediolateral = ± 1.0; dorsoventral = −2.0 (SEZ). Anesthesia was terminated by subcutaneous administration of atipamezole (2.5 mg kg^−1^, Janssen), flumazenil (0.5 mg kg^−1^, Hexal) and buprenorphine (0.1 mg kg^−1^, Essex). Mice were euthanized for analysis 2 weeks after WM/CC injection, or at 5, 14 and 28 days after SEZ injection.

#### Implantation of a transcortical window

A transcortical window was implanted in 2–5-month-old Aldh1-Cre-eGFP mice as described in ref. ^34^. Cranial bone (Ø = 3 mm) (−2.0 mm posterior, −1.5 mm lateral from bregma) was removed. A punch biopsy (Ø = 3 mm, Miltex) was inserted to a depth of 1.5 mm and cortical tissue was aspirated using a blunt needle (22-gauge, Braun); a transcortical window (made from a 3-mm Ø stainless steel cannula cut at 1.5 mm height and closed by a 3-mm Ø glass coverslip, Warner Instruments) was inserted.

In vivo imaging was performed with an upright microscope (Leica SP8 WLL DIVE FALCON) with a two-photon source (Insight X3 DUAL, Spectra Physics). GFP was excited by a laser tuned to 940 nm through an objective with a long working distance (3-mm working distance, water immersion, ×16 magnification and 0.8-numerical aperture, Nikon). Mice were anesthetized using an intraperitoneal injection of fentanyl (0.05 mg kg^−1^, Janssen), midazolam (5 mg kg^−1^, Roche) and medetomidine (0.5 mg kg^−1^, Fort Dodge). Body temperature was maintained at 37 °C using a monitoring system (MARTA Pad, Vigilitec). The rim of the cannula was used to define four quadrants (four fields of view (FOVs)). Each FOV was recorded on days 19, 21 and 22 (cohort 1) or on days 18, 24, 31, 41 and 45 (cohort 2) after window implantation (*n* = 5). Anesthesia was terminated with atipamezole (2.5 mg kg^−1^, Janssen), flumazenil (0.5 mg kg^−1^, Hexal) and buprenorphine (0.1 mg kg^−1^, Essex).

The data analyzed in Extended Data Fig. 8 was acquired at the Brain Research Institute, University of Zurich^35^. Recombination in Ascl1-CreERT2/tdTom mice was induced by tamoxifen injection (180 mg kg^−1^ body weight, Sigma-Aldrich) 2–3 days after window placement. The dataset—without demyelinating lesion—was screened for astrocytes that, in addition to OPCs, are recombined in the Ascl1-CreERT2/tdTom mouse line.

#### Tracking astrocytes

Aligned astrocytes (Imaris Microscopy Image Analysis v.9.7.4) were tracked throughout time points with TrackMate^53,54^. A Laplacian of Gaussian detector set with a 10-μm object diameter and a quality threshold of 20 identified the astrocytes. A linear assignment problem tracker set with a 10-μm max frame-to-frame linking, two-frame gap closing and 15-μm gap closing linking tracked the astrocytes through the time points. XYZ displacement, Z displacement and duration were extracted. XYZ and Z displacement were divided by the duration to create the average displacement per time frame.

### Tissue immunohistochemistry

#### Mouse sections

Mice were perfused transcardially under anesthesia with ketamine (100 mg kg^−1^) and xylazine (10 mg kg^−1^) with 5 ml 1× PBS followed by 50 ml 4% paraformaldehyde (PFA). Brains were postfixed with 4% PFA for 1 h at room temperature (RT). Brains were cut into 40-µm-thick sagittal sections using a vibrating microtome. For immunostaining, sections were incubated for 30 min in blocking solution (PBS with 2% BSA and 0.5% Triton X-100). The following primary antibodies were used and incubated for 48 h at 4 °C: chicken anti-GFP (1:300 dilution, Aves Labs); rat anti-RFP (1:500 dilution; Rockland); mouse anti-GFAP (1:500 dilution, Sigma-Aldrich); rabbit anti-Sox9 (1:1,000 dilution, Merck Millipore); goat anti-Sox9 (1:1,000 dilution, R&D Systems); rabbit anti-HMGB2 (1:1,000 dilution, Abcam); rabbit anti-RPA2 (1:250 dilution, Abcam); rabbit anti-THBS4 (1:100 dilution, Abcam); rabbit anti-S100 alpha 6 (1:500 dilution, Abcam); mouse anti-RACK1 (1:500 dilution, BD Biosciences); and rat anti-CD31 (1:100 dilution, BD Biosciences). After washing (1× PBS, 3 × 10 min at RT), secondary antibodies were incubated at 4 °C for 24 h: anti-chicken Alexa Fluor 488 (1:500 dilution, Jackson ImmunoResearch); anti-rat Alexa Fluor 546 (1:500 dilution, Thermo Fisher Scientific); anti-rabbit Alexa Fluor 594 (1:500 dilution, Thermo Fisher Scientific); anti-rabbit IgG (H+L) Alexa Fluor 488 (1:500 dilution, Thermo Fisher Scientific); anti-goat Alexa Fluor 488 (1:500 dilution, Thermo Fisher Scientific); anti-mouse IgG Alexa Fluor 488 (1:500 dilution, Thermo Fisher Scientific); anti-mouse IgG Alexa Fluor 594 (1:500 dilution, Thermo Fisher Scientific) and anti-mouse IgG Alexa Fluor 647 (1:500 dilution, Thermo Fisher Scientific). For nuclear staining: sections were incubated for 15 min at RT with 4′,6-diamidino-2-phenylindole (DAPI) (0.1 mg ml^−1^, cat. no. D9564-10 mg, Sigma-Aldrich). 5-EdU incorporation was visualized with the ClickiT EdU Alexa Fluor 647 Imaging Kit (Thermo Fisher Scientific).

#### Human sections

Individual autopsy samples of human cerebral cortex were collected and fixed with 4% PFA for 72 h. For ethical reasons, all specimens were anonymized, thereby leaving no possibility to trace back specific individuals. Inclusion criteria were a minimum age of 18 years and minimal autolytic changes of brain tissue. The histopathological state of tissue was examined with GFAP and Iba1 immunolabeling; only samples without any signs of reactive gliosis were used. Collection and use were carried out in accordance with the legal guidelines of the Government of Upper Bavaria (BayKrG Art. 27 Abs. 4) and approved by the ethics committee of LMU Munich.

Tissue was embedded in 4% agarose; 50-µm-thick sections were prepared using a Leica VT1000S Vibratome. For immunostaining, sections were pretreated with blocking solution (PBS, 2% BSA, 0.5% Triton X-100) for 45 min at RT and incubated with primary antibodies: anti-GFAP (1:250 dilution, Sigma-Aldrich); anti-IBA1 (1:500 dilution, Wako); or S100α6 (1:500 dilution, Abcam) overnight at 4 °C. After washing with PBS (3× 10 min at RT), sections were incubated with secondary antibodies: anti-mouse IgG1 Alexa Fluor 488 (1:500 dilution, Thermo Fisher Scientific) and anti-rabbit IgG Cy3 (1:500 dilution, Jackson ImmunoResearch) for 90 min at RT. All sections were incubated for 15 min at RT with DAPI (0.1 mg ml^−1^) for nuclear labeling.

### RNAscope

The RNAscope Multiplex Fluorescent Reagent Kit v2 (ACDBio) was used according to the manufacturer’s directions. For hybridization, the following probes were obtained from ACDBio: *Slc1a3* (Probe-Mm-*Slc1a3*-C2 Manual Assay); *Gria2* (Probe-Mm-*Gria2*-O1-C3 Manual Assay); *Vim* (Probe-Mm-*Vim* Manual Assay); *Hes6* (Probe-Mm-*Hes6* Manual Assay); *Ascl1* (Probe-Mm-*Ascl1*-CDS-C3 Manual Assay); *Lima1* (Probe-Mm-*Lima1* Manual Assay) and *Slc7a10* (Probe-Mm-*Slc7a10* Manual Assay). For immunostaining, sections were incubated in 4% PFA for 10 min at RT and washed twice in 1× PBS before adding blocking solution. If no immunostaining was needed, DAPI (1:1,000) was added for 10 min at RT.

### Image acquisition, processing, quantification and statistical analysis

Confocal microscopy was performed with a ZEISS LSM 710 microscope using the ZEN software (black edition, v.2.3 SP1, ZEISS) and a Leica TCS SP8 X microscope using the LAS X software (v.3.5.7.23225, Leica). Images were acquired with a ×25/0.80 and 40×/1.3 objective. Image processing was performed using ImageJ (v.2.14.0/1.54f, NIH).

For immunostaining, a minimum of 2–3 sections per animal were analyzed. In each section, the region of interest was selected and the number of positive cells in all individual z-planes of an optical z-stack was quantified. To account for variations in area size and section thickness, total cell numbers were normalized.

For RNAscope analysis, one section per animal was analyzed using QuPath^55^. Cell detection was conducted with the ‘cell detection’ module using DAPI to detect the region of interest. Using the ‘subcellular detection’ module, the quantity of distinct punctate dots was counted in each cell. A threshold of more than six estimated dots per cell for each candidate and more than 11 estimated dots per cell for *Slc1a3* was established.

### scRNA-seq

Cells from GM cortices (13 male mice, bilateral sampling using a punch biopsy (∅ = 0.25 cm) for three 10× reactions, three independent experiments (*n*_1_ = 3 mice; *n*_2_ = 3 mice; *n*_3_ = 7 mice)), WM from CC (29 male mice, bilateral sampling for five 10× reactions, four independent experiments (*n*_1_ = 6 mice; *n*_2_ and *n*_3_ = 10 mice; *n*_4_ = 6 mice; *n*_5_ = 7 mice), SEZ (15 male mice, bilateral sampling for two 10× reactions, two independent experiments (*n*_1_ = 7 mice; *n*_2_ = 8 mice)) and from the cerebellar WM (12 male mice for two 10× reactions, two independent experiments (*n*_1_ = 6 mice; *n*_2_ = 6 mice)) were isolated from C57BL/6J male mice using the Papain Dissociation System (Worthington Biochemical) followed by the Dead Cell Removal kit (cat. no. 130-090-101, Miltenyi Biotec). Incubation with dissociating enzyme was performed for 60 min. Myelin debris was removed from the WM samples using Myelin Removal Beads II (Miltenyi Biotec). Cells were suspended in 1× PBS with 0.04% BSA for a final concentration of 1,000 cells µl^−1^. Single-cell suspensions were processed using the Single Cell 3′ Reagent Kits v3.1. Illumina libraries were sequenced with the HiSeq 4000 or NovaSeq 6000 system after quality assessment with Bioanalyzer (Agilent Technologies), with an average read depth of 30,000 raw reads per cell.

#### Dissection of cerebellar WM

Parasagittal cerebellar slices were obtained by placing cerebella into an adult mouse brain matrix slicer, yielding sagittal sections with 1.0-mm intervals. Examination under a stereomicroscope was conducted, leading to the selection of slices exhibiting detectable WM on both sides. The WM arbor vitae facing the bases of the lobules (deep WM) and in the lobules was isolated. The GM of the cerebellar nuclei situated at deep positions in the cerebellar hemispheres and the lobular GM were excised. The lobular WM posed challenges because of its slender nature, affecting dissection resolution and probably contributing to a degree of GM contamination.

### Single-cell/nucleus analysis

scRNA-seq reads were aligned against the mm10 mouse genome (build v.1.2.0 and v.2020A from 10x Genomics) using CellRanger v.6.0.1 with default settings. Datasets were processed using CellBender to remove technical noise and ambient RNA, thereby enhancing data quality and accuracy^20^. Subsequent analysis was performed using the Seurat pipeline v.4.3 (ref. ^56^) on R v.4.2.1 (http://www.R-project.org/). Quality control of cells was done by following the recommendations^57^, selecting cells with at least 350 genes and a maximum of 15% mitochondrial fraction. Doublets were removed by excluding cells with more than 5,000 genes. Gene expression values for each cell were divided by the total number of transcripts and multiplied by 10,000. Values were then log-transformed using log1p using the NormalizeData() function. Genes were scaled and centered using the ScaleData() function. We used Harmony (v.1.0)^58^ in the Seurat workflow with default parameters to integrate different datasets. For cluster analysis, we constructed a shared nearest neighbor graph based on Harmony embeddings using the FindNeighbors() function as input for the SLM algorithm, implemented through the FindClusters() function in Seurat (see the main text for the exact number of dimensions used in each analysis). Cluster-specific marker genes were identified by comparing the cells of each cluster to cells from all other clusters using the Wilcoxon rank-sum test implemented in the Seurat function FindAllMarkers(). Clusters were manually annotated based on marker gene expression, spatial transcriptome mapping, and by using the online database (http://mousebrain.org). GO analysis was performed using Metascape (https://metascape.org)^59^. To calculate the single-cell velocity^60^ of gene expression from exonic and intronic reads, we used the Python (v.3.8.8) packages Scanpy (v.1.9.3) and Scvelo (v.0.2.5)^61^ with projection time eq. 1, gamma fit on 2% quantiles of expression values, and slope calculations smoothed over 25 nearest cells. Velocity values, logarithmically scaled and multiplied, were used to highlight interesting features, then smoothed over 200 cells and overlaid as arrows onto the *t*-SNE projection of the clustered scRNA-seq data. To analyze cell–cell communication networks and infer interaction patterns from the scRNA-seq data, we used CellChat^62^.

### Visium spatial gene expression

The brain from a male C57BL/6J mouse was embedded and snap-frozen in an isopentane and liquid nitrogen bath, according to the 10x Genomics protocol. During cryosectioning (CryoStar NX50, Thermo Fisher Scientific), a 10-µm-thick sagittal section was collected. The tissue was stained with hematoxylin and eosin staining and imaged with a ZEISS Axio Imager M2 Microscope (×10 objective). Libraries were prepared according to the Visium Spatial Gene Expression Reagent Kits with an 18-min permeabilization time and sequenced on an Illumina HiSeq 1500 system with a paired-end flow cell (high-output). The sequencing depth achieved was 65,433 mean reads per spot. Sequencing was performed in the Laboratory for Functional Genome Analysis.

### Visium spatial gene expression analysis

Data were mapped against the mouse reference genome mm10 (GENCODE vM23/Ensembl 98; builds v.1.2.0 and v.2020A from 10x Genomics) with Space Ranger v.1.2.2. The dataset was analyzed, followed by quality checking of the Seurat pipeline (Seurat v.3.2)^56^. To infer the spatial location of clusters, the single-cell/nucleus RNA-seq datasets were integrated with the Visium spatial transcriptomic datasets. We applied an ‘anchor’-based integration workflow in Seurat, which enables the probabilistic transfer of annotations from a reference to a query set. The spatial reference and lineage datasets were normalized using the SCTransform() function, which builds regularized negative binomial models of gene expression. We performed dimensionality reduction using the RunPCA() function and performed label transfer using the functions FindTransferAnchors() and TransferData(). This procedure outputs, for each spatial spot, a probabilistic classification for each of the scRNA-seq-derived cell states. We added these predictions as a new assay in the Seurat object for visualization using the SpatialFeaturePlot() function.

### MERFISH spatial dataset analysis

The publicly available multiplexed error-robust fluorescence in situ hybridization (MERFISH) dataset was produced using the Vizgen MERSCOPE system and analyzed according to the Seurat pipeline (Seurat v.3.2)^56^. As with the scRNA-seq experiments, we used SCTransform-based normalization and performed dimensional reduction and clustering. The gene panel consists of 483 gene targets, representing known markers that allowed us to discriminate cell types.

### Statistics, reproducibility and text editing

Randomly selected mice were assigned to different experimental groups. No further randomization was applied during data collection. Investigators were blinded to group assignment during the experiments and data analysis.

All statistical tests were performed with Prism v.8.4.3 (GraphPad Software). Statistical significance was defined as *P < 0.05, **P < 0.01 and ***P < 0.001. All biological replicates (*n*) were derived from at least three independent experiments. All column graphs are expressed as the median ± s.e.m. The normality of the distribution of data points was verified using the Shapiro–Wilk test. The Brown–Forsythe test was used to access the equality of group variances.

No statistical methods were used to predetermine sample sizes but our sample sizes are similar to those reported in previous publications^17,29^. No animals or data points were excluded from the analyses.

Micrographs shown in the following figures are representative of biological replicates: Fig. 6g (*n* = 3 donors); Fig. 7e (*n* = 5); Extended Data Fig. 3b (*n* = 4); Extended Data Fig. 3c (*n* = 4); Extended Data Fig. 7c (*n* = 3); Extended Data Fig. 7d (*n* = 3); and Extended Data Fig. 7e (*n* = 5). For Fig. 8e, the analysis was performed on the following sample sizes (animals): *n* = 3 for the 3-month and 6-month time points; *n* = 4 for the 4-month time point; *n* = 5 for the 5-month time point; *n* = 6 for the 1-month time point; and *n* = 7 for the 2-month time point. The analysis in the GM in Fig. 7c was performed with *n* = 2 for the 2-month and 5-month time points, and *n* = 3 for the 1-month, 3-month, 4-month and 6-month time points. The analysis in the WM was performed in *n* = 2 for the 3-month and 6-month time points, *n* = 3 for the 4-month time point, *n* = 4 for the 5-month time point, *n* = 6 for the 1-month time point and *n* = 8 animals for the 2-month time point.

We used ChatGPT (OpenAI) to assist in language editing and text refinement.

### Reporting summary

Further information on research design is available in the Nature Portfolio Reporting Summary linked to this article.

## Online content

Any methods, additional references, Nature Portfolio reporting summaries, source data, extended data, supplementary information, acknowledgements, peer review information; details of author contributions and competing interests; and statements of data and code availability are available at 10.1038/s41593-025-01878-6.

## Supplementary information

Reporting Summary

Supplementary Table 1 List of genes used for calculating cell type-specific scores.

Supplementary Table 2 **Differential gene expression analysis between astrocyte clusters**. This analysis is based on the astrocyte clusters presented in Fig. 2a, left. Statistical significance was tested using a two-sided Mann–Whitney *U*-test with Bonferroni correction for multiple comparisons. All *P*values are provided in the table.

Supplementary Table 3 **Differential gene expression analysis between astrocytes in layer 1, gray and white matter**. This analysis compares gene expression among astrocytes located in layer 1, gray and white matter as presented in Fig. 2a, right. Results are visualized in Fig. 3a. Statistical significance was assessed using a two-sided Mann–Whitney *U*-test with Bonferroni correction for multiple comparisons. All *P*values are included in the table.

Supplementary Table 4 **Gene ontology analysis of astrocytes in layer 1, gray matter and white matter**. This analysis highlights enriched gene ontologies among astrocytes located in L1, gray matter and white matter. Results are visualized in Fig. 3e.

Supplementary Table 5 List of genes used for calculating functional scores.

Supplementary Table 6 **Differential gene expression analysis between white matter astrocyte clusters 4 and 5**. This analysis compares gene expression between white matter astrocytes in cluster 4 to those in cluster 5, as shown in Fig. 4a. Results are visualized in Fig. 4b. Statistical significance was evaluated using a two-sided Mann-Whitney U test with Bonferroni correction for multiple comparisons. All *P*-values are provided in the table.

Supplementary Table 7 **Gene ontology analysis of white matter astrocytes in clusters 4 and 5**. This analysis highlights enriched gene ontologies among white matter astrocytes in clusters 4 and 5, as presented in Fig. 4a. Results are visualized in Fig. 4d.

Supplementary Table 8 **List of genes identified through RNA velocity analysis**. This table provides a list of dynamically regulated genes along the differentiation trajectory from cluster 5 to cluster 4, identified using RNA velocity analysis. Results are visualized in Fig. 8c.

## Source data

Source Data Fig. 3 Statistical source data.

Source Data Fig. 7 Statistical source data.

Source Data Fig. 8 Statistical source data.

Source Data Extended Data Fig. 3 Statistical source data.

Source Data Extended Data Fig. 7 Statistical source data.

Source Data Extended Data Fig. 9 Statistical source data.

## Data Availability

The mouse reference genome mm10 (https://www.10xgenomics.com/support/software/cell-ranger/downloads/cr-ref-build-steps) was used for data alignment. All datasets generated can be accessed at http://bocchilab.ch/Bocchi_et_al_2024. Raw data are available from the Sequence Read Archive under accession no. PRJNA1125165. Publicly available gene expression data used for cluster annotation can be accessed at the Mouse Brain Atlas (http://mousebrain.org/). The Visium spatial transcriptomic dataset used for the cerebellum analysis is provided by 10x Genomics (https://support.10xgenomics.com/spatial-gene-expression/datasets; mouse brain serial section 2, sagittal-posterior). The human single-nucleus dataset was generated and obtained from Roche and downloaded with their permission from the European Genome-phenome Archive (https://ega-archive.org, accession no. EGAD00001009169). For our analysis, we only used the data from control patients (nos. 86, 98, 107, 117, 121, 126, 131, 133, 135, 139, 140, 144 and 145). The Visium spatial transcriptomic dataset for the human cortex was obtained from the study by Maynard et al.^32^. The raw data are publicly available from the LIBD Globus endpoint ‘jhpce#HumanPilot10x’ listed at http://research.libd.org/globus. The MERFISH spatial dataset was provided by Vizgen (https://info.vizgen.com/mouse-brain-map; MERFISH Mouse Brain Receptor Map). Source data are provided with this paper.

## Code availability

No original code was used in this study. The code and pipelines used in this study can be provided upon reasonable request.
