# Supplementary material for: Astrocyte heterogeneity reveals region-specific astrogenesis in the white matter
Source: Nat Neurosci. 2025 Feb 24;28(3):457–69. doi: 10.1038/s41593-025-01878-6 (PMC11893471; doi:10.1038/s41593-025-01878-6)
Supplement: Supplementary file 1 — Reporting Summary [file 41593_2025_1878_MOESM1_ESM.pdf]

nature portfolio

Corresponding author(s): Riccardo Bocchi, Magdalena Götz, Judith Fischer-Sternjak

Last updated by author(s): Dec 16, 2024

Reporting Summary

Nature Portfolio wishes to improve the reproducibility of the work that we publish. This form provides structure for consistency and transparency in reporting. For further information on Nature Portfolio policies, see our Editorial Policies and the Editorial Policy Checklist.

Statistics

For all statistical analyses, confirm that the following items are present in the figure legend, table legend, main text, or Methods section.

n/a	Confirmed	
☐	☒	The exact sample size (*n*) for each experimental group/condition, given as a discrete number and unit of measurement
☐	☒	A statement on whether measurements were taken from distinct samples or whether the same sample was measured repeatedly
☐	☒	The statistical test(s) used AND whether they are one- or two-sided<br>*Only common tests should be described solely by name; describe more complex techniques in the Methods section.*
☒	☐	A description of all covariates tested
☐	☒	A description of any assumptions or corrections, such as tests of normality and adjustment for multiple comparisons
☐	☒	A full description of the statistical parameters including central tendency (e.g. means) or other basic estimates (e.g. regression coefficient) AND variation (e.g. standard deviation) or associated estimates of uncertainty (e.g. confidence intervals)
☐	☒	For null hypothesis testing, the test statistic (e.g. *F*, *t*, *r*) with confidence intervals, effect sizes, degrees of freedom and *P* value noted<br>*Give P values as exact values whenever suitable.*
☒	☐	For Bayesian analysis, information on the choice of priors and Markov chain Monte Carlo settings
☒	☐	For hierarchical and complex designs, identification of the appropriate level for tests and full reporting of outcomes
☒	☐	Estimates of effect sizes (e.g. Cohen's *d*, Pearson's *r*), indicating how they were calculated

Our web collection on statistics for biologists contains articles on many of the points above.

Software and code

Policy information about availability of computer code

Data collection	Leica SP8 WLL DIVE FALCON 2Photon microscope, Zeiss Laser-scanning confocal microscope LSM710, Zeiss Axio Observer Z1 epifluorescence microscope & Zen software (v2.3), Illumina HiSeq1500, Illumina NovaSeq6000, Leica SP8X WLL upright confocal microscope & Leica LAS X software version 3.5.7.23225
Data analysis	Confocal images were analyzed with ImageJ software (version 2.14.0/1.54f) and QuPath software (version 0.50-x64).<br>Statistical analysis was performed using GraphPad Prism (v 8.4.3).<br>Illumina sequencing libraries were sequenced with HiSeq 4000 or NovaSeq 6000 after quality assessment with the Bioanalyzer (Agilent) with an average read depth of 30000 raw reads per cell. Alignment was performed using Cell Ranger (v6.0.1). All datasets were processed using CellBender (v0.3.0) to remove technical noise and ambient RNA. R(v4.2.1) with Seurat package (v4.3.0) was used to analyze st- & sc-RNA-seq datasets. Harmony package (v1.2.1) was used to integrated the different datasets. For RNA-velocity and PAGAplot analysis we used Python (v3.8.8) packages Scanpy (v1.9.3) and Scvelo (v0.2.5). Gene ontology analysis was performed using the online application Metascape. CellChat package (v2) was used to perform the intercellular communication networks analysis.

For manuscripts utilizing custom algorithms or software that are central to the research but not yet described in published literature, software must be made available to editors and reviewers. We strongly encourage code deposition in a community repository (e.g. GitHub). See the Nature Portfolio guidelines for submitting code & software for further information.

Data

Policy information about availability of data

All manuscripts must include a data availability statement. This statement should provide the following information, where applicable:

- Accession codes, unique identifiers, or web links for publicly available datasets
- A description of any restrictions on data availability
- For clinical datasets or third party data, please ensure that the statement adheres to our policy

The mouse reference genome mm10 [https://www.10xgenomics.com/support/software/cell-ranger/downloads/cr-ref-build-steps] was used for the data alignment. All datasets generated for the current study can be browsed at http://bocchilab.ch/Bocchi_et_al_2024. Raw data are available in the Sequence Read Archive (SRA) under the accession number PRJNA1125165. Publicly available gene expression data used for cluster annotation can be accessed at the Mouse Brain Atlas (http://mousebrain.org/genesearch.html). The Visium spatial transcriptomic dataset on the mouse brain sagital section, used for the cerebellum analysis, is provided by 10x Genomics (https://support.10xgenomics.com/spatial-gene-expression/datasets; Mouse Brain Serial Section 2, Sagittal-Posterior). The human single nuclei dataset was generated and obtained from Roche and downloaded with their permission from the European Genome-phenome Archive (EGA, https://ega-archive.org, accession number EGAD00001009169). For our analysis, we only used the data from control patients (i.e., 86, 98, 107, 117, 121, 126, 131, 133, 135, 139, 140, 144, and 145). The Visium spatial transcriptomic dataset on the human cortex was obtained from the study by Maynard et al.42. The raw data are publicly available from the Globus endpoint 'jhpce#HumanPilot10x' that is also listed at http://research.libd.org/globus. The Merfish spatial dataset on the mouse brain coronal section is provided by Vizgen (https://info.vizgen.com/mouse-brain-map; MERFISH Mouse Brain Receptor Map).

Research involving human participants, their data, or biological material

Policy information about studies with human participants or human data. See also policy information about sex, gender (identity/presentation), and sexual orientation and race, ethnicity and racism.

Reporting on sex and gender	Sex and gender were not considered in study design. The biomaterials were made available for research purposes only after double-coding anonymization, so we have no information regarding sex and gender of the patients.
Reporting on race, ethnicity, or other socially relevant groupings	All tissue samples were anonymized for ethical reasons, thereby leaving no possibility to trace back specific deceased subjects, so we have no information regarding population characteristics besides a minimum subject age ≥ 18 years.
Population characteristics	Postmortem brain tissue samples were collected during the course of a standard forensic autopsy at the Institute of Forensic Medicine at the Ludwig-Maximilians University Munich, Germany. After completion of court-ordered examinations, specimens of contusion areas within the parietal lobe of cerebral cortex were obtained. All tissue samples were anonymized for ethical reasons, thereby leaving no possibility to trace back specific deceased subjects, so we have no information regarding population characteristics besides a minimum subject age ≥ 18 years.
Recruitment	For analysis of postmortem brain tissue, samples were collected after completion of court-ordered examination at the Institute of Forensic Medicine (Ludwig-Maximilians University in Munich, Germany) according to the following inclusion criteria: (I) the order of a forensic autopsy by the local prosecutor, (II) older than 18 years of age and (III) the minimal autolytic changes of the brain tissue.
Ethics oversight	The collection of postmortem tissue samples and their usage for research purpose occurred in accordance to the legal guidelines of Government of Upper Bavaria (BayKrG Art. 27 Abs. 4) and was approved from the Ethical review board at Ludwig-Maximilian-University Munich, Germany (DNO 087-13).

Note that full information on the approval of the study protocol must also be provided in the manuscript.

Field-specific reporting

Please select the one below that is the best fit for your research. If you are not sure, read the appropriate sections before making your selection.

☒ Life sciences ☐ Behavioural & social sciences ☐ Ecological, evolutionary & environmental sciences

For a reference copy of the document with all sections, see nature.com/documents/nr-reporting-summary-flat.pdf

Life sciences study design

All studies must disclose on these points even when the disclosure is negative.

Sample size	Sample sizes were determined without the use of statistical approaches; instead, they were chosen by referencing prior studies with similar experimental setups: Ohlig et al., 2021; Koupourtidou et al., 2024.
Data exclusions	No data were excluded.
Replication	st-RNA-seq experiment was performed once. All sc-RNA-seq experiments were performed with at least 2 replicates. More details about the replication and number of animals used for each of these experiments can be found in the methods section.

	All experiments were repeated at least 2x and the results could be replicated each time. The number of replicates for each experiment and sample sizes are provided in the figure legends and the methods section.
Randomization	Mice were chosen in a random manner and distributed among various experimental groups. There was no further randomization during the process of data collection. Human postmortem brain tissue was chosen in a random manner.
Blinding	The data collection and analysis process was conducted without the investigators knowing the group assignments (blinded). After the analysis was finished, the group assignments were disclosed.

Behavioural & social sciences study design

All studies must disclose on these points even when the disclosure is negative.

Study description	*Briefly describe the study type including whether data are quantitative, qualitative, or mixed-methods (e.g. qualitative cross-sectional, quantitative experimental, mixed-methods case study).*
Research sample	*State the research sample (e.g. Harvard university undergraduates, villagers in rural India) and provide relevant demographic information (e.g. age, sex) and indicate whether the sample is representative. Provide a rationale for the study sample chosen. For studies involving existing datasets, please describe the dataset and source.*
Sampling strategy	*Describe the sampling procedure (e.g. random, snowball, stratified, convenience). Describe the statistical methods that were used to predetermine sample size OR if no sample-size calculation was performed, describe how sample sizes were chosen and provide a rationale for why these sample sizes are sufficient. For qualitative data, please indicate whether data saturation was considered, and what criteria were used to decide that no further sampling was needed.*
Data collection	*Provide details about the data collection procedure, including the instruments or devices used to record the data (e.g. pen and paper, computer, eye tracker, video or audio equipment) whether anyone was present besides the participant(s) and the researcher, and whether the researcher was blind to experimental condition and/or the study hypothesis during data collection.*
Timing	*Indicate the start and stop dates of data collection. If there is a gap between collection periods, state the dates for each sample cohort.*
Data exclusions	*If no data were excluded from the analyses, state so OR if data were excluded, provide the exact number of exclusions and the rationale behind them, indicating whether exclusion criteria were pre-established.*
Non-participation	*State how many participants dropped out/declined participation and the reason(s) given OR provide response rate OR state that no participants dropped out/declined participation.*
Randomization	*If participants were not allocated into experimental groups, state so OR describe how participants were allocated to groups, and if allocation was not random, describe how covariates were controlled.*

Ecological, evolutionary & environmental sciences study design

All studies must disclose on these points even when the disclosure is negative.

Study description	*Briefly describe the study. For quantitative data include treatment factors and interactions, design structure (e.g. factorial, nested, hierarchical), nature and number of experimental units and replicates.*
Research sample	*Describe the research sample (e.g. a group of tagged Passer domesticus, all Stenocereus thurberi within Organ Pipe Cactus National Monument), and provide a rationale for the sample choice. When relevant, describe the organism taxa, source, sex, age range and any manipulations. State what population the sample is meant to represent when applicable. For studies involving existing datasets, describe the data and its source.*
Sampling strategy	*Note the sampling procedure. Describe the statistical methods that were used to predetermine sample size OR if no sample-size calculation was performed, describe how sample sizes were chosen and provide a rationale for why these sample sizes are sufficient.*
Data collection	*Describe the data collection procedure, including who recorded the data and how.*
Timing and spatial scale	*Indicate the start and stop dates of data collection, noting the frequency and periodicity of sampling and providing a rationale for these choices. If there is a gap between collection periods, state the dates for each sample cohort. Specify the spatial scale from which the data are taken*
Data exclusions	*If no data were excluded from the analyses, state so OR if data were excluded, describe the exclusions and the rationale behind them, indicating whether exclusion criteria were pre-established.*
Reproducibility	*Describe the measures taken to verify the reproducibility of experimental findings. For each experiment, note whether any attempts to repeat the experiment failed OR state that all attempts to repeat the experiment were successful.*
Randomization	*Describe how samples/organisms/participants were allocated into groups. If allocation was not random, describe how covariates were controlled. If this is not relevant to your study, explain why.*

Blinding

Describe the extent of blinding used during data acquisition and analysis. If blinding was not possible, describe why OR explain why blinding was not relevant to your study.

Did the study involve field work? ☐ Yes ☐ No

Field work, collection and transport

Field conditions

Describe the study conditions for field work, providing relevant parameters (e.g. temperature, rainfall).

Location

State the location of the sampling or experiment, providing relevant parameters (e.g. latitude and longitude, elevation, water depth).

Access & import/export

Describe the efforts you have made to access habitats and to collect and import/export your samples in a responsible manner and in compliance with local, national and international laws, noting any permits that were obtained (give the name of the issuing authority, the date of issue, and any identifying information).

Disturbance

Describe any disturbance caused by the study and how it was minimized.

Reporting for specific materials, systems and methods

We require information from authors about some types of materials, experimental systems and methods used in many studies. Here, indicate whether each material, system or method listed is relevant to your study. If you are not sure if a list item applies to your research, read the appropriate section before selecting a response.

Materials & experimental systems

n/a	Involved in the study
☐	☒ Antibodies
☒	☐ Eukaryotic cell lines
☒	☐ Palaeontology and archaeology
☐	☒ Animals and other organisms
☒	☐ Clinical data
☒	☐ Dual use research of concern
☒	☐ Plants

Methods

n/a	Involved in the study
☒	☐ ChIP-seq
☒	☐ Flow cytometry
☒	☐ MRI-based neuroimaging

Antibodies

Antibodies used

1) anti-GFP, Aves Labs, cat. number: GFP-1020, lot number: GFP3717982, chicken polyclonal, dilution: 1:300
2) anti-HMGB2, Abcam, cat. number: ab67282, lot number: GR3332496-7, rabbit polyclonal, dilution: 1:1000
3) anti-RFP, ROCKLAND, cat. number: 600-401-379, lot number: 46510, rabbit Polyclonal, dilution: 1:500
4) anti-RPA32/RPA2 [EPR2877Y], Abcam, cat. number: ab76420, lot number: GR34309003-6, rabbit monoclonal, dilution: 1:250
5) anti S100 alpha 6/PRA [EPR13084-69], Abcam, cat. number: ab181975, lot number: GR3438351-2, rabbit monoclonal, dilution: 1:500
6) anti-Sox9, Merck Millipore, cat. number: ab5535, lot number: 3856123, rabbit polyclonal, dilution: 1:1000
7) anti-THBS4 [EPR22922-232], Abcam, cat. number: ab263898, lot number: GR3388834-4, rabbit monoclonal, dilution: 1:100
8) anti-GFAP, Sigma Aldrich, cat. number: G3893, lot number: 0000180345, mouse IgG1 monoclonal, dilution: 1:250
9) anti-Iba1, FUJIFILM Wako Pure Chemical Corporation, cat. number 019-19741, lot number SKP3626, rabbit polyclonal, dilution: 1:500
10) anti-RACK1, BD Biosciences, cat. number 610178, lot number 9183699, mIgG1 monoclonal, dilution 1:500
11) anti-Sox9, R&D Systems, cat. number AF3075, lot number WIL 0522101, goat polyclonal, dilution 1:1.000
12) anti-CD31, BD Biosciences, cat. number 550274, lot number 1025824, rat IgG 2a, monoclonal, dilution 1:100

1’) anti-chicken IgY (IgG) (H+L) Alexa Fluor 488, Jackson Immuno Research, cat. number: 703-545-155, lot number: 156558, dilution: 1:500
2’) anti-rabbit IgG (H+L) Alexa Fluor A594, Invitrogen, cat. number: A21207, lot number: 2313074, dilution: 1:500
3’) anti-rabbit Alexa IgG (H+L) Fluor A647, Invitrogen, cat. number: A31573, lot number: 2420695, dilution: 1:500
4’) anti-rat IgG (H+L) Alexa Fluor A546, Invitrogen, cat. number: A11081, lot number: 2304272, dilution: 1:500
5’) anti-mouse IgG1 Alexa Fluor 488, Invitrogen, cat. number A21121, lot number 2465091, dilution: 1:500
6’) anti-rabbit IgG (H+L) Cy3, Jackson ImmunoResearch, cat. number 711-165-152, lot no.159919, dilution: 1:500
7’) anti-goat IgG (H+L) Alexa Fluor 488, Invitrogen, cat. number: A11055, lot number: 2411589, dilution 1:500
8') anti-mouse IgG (H+L) Alexa Fluor 594, Invitrogen, cat. number A121203, lot number: 2474956, dilution 1:500
9') anti-mouse IgG(H+L) Alexa Fluor 647, Invitrogen, cat. number A32787, lot number: YB363609, dilution 1:500
10') anti-rabbit IgG(H+L) Alexa Fluor 488, Invitrogen, cat. number A21206, lot number: 2668665, dilution 1:500

Validation

1) anti-GFP has been validated previously in mouse brain tissue (Ohlig et al., 2021; PMID: 34549820)
2) anti-HMGB2 has been validated previously in mouse brain tissue (Kimura et al., 2018; PMID: 28771884)

3) anti-RFP has been validated previously in mouse brain tissue (Zhang et al., 2022; PMID: 35705049)
4) anti-RPA32/RPA2 has been validated (Bienkowska-Haba et al., 2020; PMID: 31748387)
5) anti-S100a6 has been validated previously in mouse brain tissue (Kjell et al., 2020; PMID: 32032526)
6) anti-Sox9 has been validated previously in mouse brain tissue (Ohlig et al., 2021; PMID: 34549820)
7) anti-THBS4 has been validated previously in mouse tissue (Best et al., 2021; PMID: 33480357)
8) anti-GFAP has been validated in human brain tumors (Weng et al., 2019, PMID: 30982771) and in human iPSC-derived astrocytes (Li et al., 2018, PMID: 30075130)
9) anti-Iba1 has been validated in human brain tissue and glioma cell lines (Kuan et al., 2016, PMID: 27632900; Dekens et al., 2017, PMID: 27716662; Keane et al., 2021, PMID: 34485907)
10) anti-RACK1 has been validated previously in mouse brain tissue (Oudart et al., 2023, PMID: 37126448)
11) anti-Sox9 has been validated previously in mouse brain tissue (Sofroniew et al., 2022, PMID: 36171203)
12) anti-CD31 has been validated previously in mouse brain tissue (Wittmann et al., 2015, PMID: 26337286)

Eukaryotic cell lines

Policy information about cell lines and Sex and Gender in Research

Cell line source(s)	*State the source of each cell line used and the sex of all primary cell lines and cells derived from human participants or vertebrate models.*
Authentication	*Describe the authentication procedures for each cell line used OR declare that none of the cell lines used were authenticated.*
Mycoplasma contamination	*Confirm that all cell lines tested negative for mycoplasma contamination OR describe the results of the testing for mycoplasma contamination OR declare that the cell lines were not tested for mycoplasma contamination.*
Commonly misidentified lines (See ICLAC register)	*Name any commonly misidentified cell lines used in the study and provide a rationale for their use.*

Palaeontology and Archaeology

Specimen provenance	*Provide provenance information for specimens and describe permits that were obtained for the work (including the name of the issuing authority, the date of issue, and any identifying information). Permits should encompass collection and, where applicable, export.*
Specimen deposition	*Indicate where the specimens have been deposited to permit free access by other researchers.*
Dating methods	*If new dates are provided, describe how they were obtained (e.g. collection, storage, sample pretreatment and measurement), where they were obtained (i.e. lab name), the calibration program and the protocol for quality assurance OR state that no new dates are provided.*

☐ Tick this box to confirm that the raw and calibrated dates are available in the paper or in Supplementary Information.

Ethics oversight	*Identify the organization(s) that approved or provided guidance on the study protocol, OR state that no ethical approval or guidance was required and explain why not.*

Note that full information on the approval of the study protocol must also be provided in the manuscript.

Animals and other research organisms

Policy information about studies involving animals; ARRIVE guidelines recommended for reporting animal research, and Sex and Gender in Research

Laboratory animals	Species: mus musculus, strains: C57BL/6J (age between 1 and 6 month), Aldh1l1-eGFP (age between 2 and 3 month), Aldh1l1Cre x eGFP (age between 2 and 5 month), Ascl1CreERT2 x tdTomato (age between 2 and 3 month)
Wild animals	no wild animals were used in this study
Reporting on sex	Both, male and female mice were used in this study, if not stated otherwise in the methods
Field-collected samples	No field-collected samples were used for this study.
Ethics oversight	All experimental procedures were performed in accordance with animal welfare policies and approved by the Government of Upper Bavaria (Germany).

Note that full information on the approval of the study protocol must also be provided in the manuscript.

Clinical data

Policy information about clinical studies

All manuscripts should comply with the ICMJE guidelines for publication of clinical research and a completed CONSORT checklist must be included with all submissions.

Clinical trial registration | *Provide the trial registration number from ClinicalTrials.gov or an equivalent agency.*

Study protocol | *Note where the full trial protocol can be accessed OR if not available, explain why.*

Data collection | *Describe the settings and locales of data collection, noting the time periods of recruitment and data collection.*

Outcomes | *Describe how you pre-defined primary and secondary outcome measures and how you assessed these measures.*

Dual use research of concern

Policy information about dual use research of concern

Hazards

Could the accidental, deliberate or reckless misuse of agents or technologies generated in the work, or the application of information presented in the manuscript, pose a threat to:

No	Yes	
☒	☐	Public health
☒	☐	National security
☒	☐	Crops and/or livestock
☒	☐	Ecosystems
☒	☐	Any other significant area

Experiments of concern

Does the work involve any of these experiments of concern:

No	Yes	
☒	☐	Demonstrate how to render a vaccine ineffective
☒	☐	Confer resistance to therapeutically useful antibiotics or antiviral agents
☒	☐	Enhance the virulence of a pathogen or render a nonpathogen virulent
☒	☐	Increase transmissibility of a pathogen
☒	☐	Alter the host range of a pathogen
☒	☐	Enable evasion of diagnostic/detection modalities
☒	☐	Enable the weaponization of a biological agent or toxin
☒	☐	Any other potentially harmful combination of experiments and agents

Plants

Seed stocks | *Report on the source of all seed stocks or other plant material used. If applicable, state the seed stock centre and catalogue number. If plant specimens were collected from the field, describe the collection location, date and sampling procedures.*

Novel plant genotypes | *Describe the methods by which all novel plant genotypes were produced. This includes those generated by transgenic approaches, gene editing, chemical/radiation-based mutagenesis and hybridization. For transgenic lines, describe the transformation method, the number of independent lines analyzed and the generation upon which experiments were performed. For gene-edited lines, describe the editor used, the endogenous sequence targeted for editing, the targeting guide RNA sequence (if applicable) and how the editor was applied.*

Authentication | *Describe any authentication procedures for each seed stock used or novel genotype generated. Describe any experiments used to assess the effect of a mutation and, where applicable, how potential secondary effects (e.g. second site T-DNA insertions, mosiacism, off-target gene editing) were examined.*

ChIP-seq

Data deposition

☐ Confirm that both raw and final processed data have been deposited in a public database such as GEO.

☐ Confirm that you have deposited or provided access to graph files (e.g. BED files) for the called peaks.

Data access links<br>*May remain private before publication.*	*For "Initial submission" or "Revised version" documents, provide reviewer access links. For your "Final submission" document, provide a link to the deposited data.*
Files in database submission	*Provide a list of all files available in the database submission.*
Genome browser session<br>(e.g. UCSC)	*Provide a link to an anonymized genome browser session for "Initial submission" and "Revised version" documents only, to enable peer review. Write "no longer applicable" for "Final submission" documents.*

Methodology

Replicates	*Describe the experimental replicates, specifying number, type and replicate agreement.*
Sequencing depth	*Describe the sequencing depth for each experiment, providing the total number of reads, uniquely mapped reads, length of reads and whether they were paired- or single-end.*
Antibodies	*Describe the antibodies used for the ChIP-seq experiments; as applicable, provide supplier name, catalog number, clone name, and lot number.*
Peak calling parameters	*Specify the command line program and parameters used for read mapping and peak calling, including the ChIP, control and index files used.*
Data quality	*Describe the methods used to ensure data quality in full detail, including how many peaks are at FDR 5% and above 5-fold enrichment.*
Software	*Describe the software used to collect and analyze the ChIP-seq data. For custom code that has been deposited into a community repository, provide accession details.*

Flow Cytometry

Plots

Confirm that:

☐ The axis labels state the marker and fluorochrome used (e.g. CD4-FITC).

☐ The axis scales are clearly visible. Include numbers along axes only for bottom left plot of group (a 'group' is an analysis of identical markers).

☐ All plots are contour plots with outliers or pseudocolor plots.

☐ A numerical value for number of cells or percentage (with statistics) is provided.

Methodology

Sample preparation	*Describe the sample preparation, detailing the biological source of the cells and any tissue processing steps used.*
Instrument	*Identify the instrument used for data collection, specifying make and model number.*
Software	*Describe the software used to collect and analyze the flow cytometry data. For custom code that has been deposited into a community repository, provide accession details.*
Cell population abundance	*Describe the abundance of the relevant cell populations within post-sort fractions, providing details on the purity of the samples and how it was determined.*
Gating strategy	*Describe the gating strategy used for all relevant experiments, specifying the preliminary FSC/SSC gates of the starting cell population, indicating where boundaries between "positive" and "negative" staining cell populations are defined.*

☐ Tick this box to confirm that a figure exemplifying the gating strategy is provided in the Supplementary Information.

Magnetic resonance imaging

Experimental design

Design type	*Indicate task or resting state; event-related or block design.*

Design specifications | *Specify the number of blocks, trials or experimental units per session and/or subject, and specify the length of each trial or block (if trials are blocked) and interval between trials.*

Behavioral performance measures | *State number and/or type of variables recorded (e.g. correct button press, response time) and what statistics were used to establish that the subjects were performing the task as expected (e.g. mean, range, and/or standard deviation across subjects).*

Acquisition

Imaging type(s) | *Specify: functional, structural, diffusion, perfusion.*

Field strength | *Specify in Tesla*

Sequence & imaging parameters | *Specify the pulse sequence type (gradient echo, spin echo, etc.), imaging type (EPI, spiral, etc.), field of view, matrix size, slice thickness, orientation and TE/TR/flip angle.*

Area of acquisition | *State whether a whole brain scan was used OR define the area of acquisition, describing how the region was determined.*

Diffusion MRI ☐ Used ☐ Not used

Preprocessing

Preprocessing software | *Provide detail on software version and revision number and on specific parameters (model/functions, brain extraction, segmentation, smoothing kernel size, etc.).*

Normalization | *If data were normalized/standardized, describe the approach(es): specify linear or non-linear and define image types used for transformation OR indicate that data were not normalized and explain rationale for lack of normalization.*

Normalization template | *Describe the template used for normalization/transformation, specifying subject space or group standardized space (e.g. original Talairach, MNI305, ICBM152) OR indicate that the data were not normalized.*

Noise and artifact removal | *Describe your procedure(s) for artifact and structured noise removal, specifying motion parameters, tissue signals and physiological signals (heart rate, respiration).*

Volume censoring | *Define your software and/or method and criteria for volume censoring, and state the extent of such censoring.*

Statistical modeling & inference

Model type and settings | *Specify type (mass univariate, multivariate, RSA, predictive, etc.) and describe essential details of the model at the first and second levels (e.g. fixed, random or mixed effects; drift or auto-correlation).*

Effect(s) tested | *Define precise effect in terms of the task or stimulus conditions instead of psychological concepts and indicate whether ANOVA or factorial designs were used.*

Specify type of analysis: ☐ Whole brain ☐ ROI-based ☐ Both

Statistic type for inference | *Specify voxel-wise or cluster-wise and report all relevant parameters for cluster-wise methods.*

(See Eklund et al. 2016)

Correction | *Describe the type of correction and how it is obtained for multiple comparisons (e.g. FWE, FDR, permutation or Monte Carlo).*

Models & analysis

n/a	Involved in the study
☐	☐ Functional and/or effective connectivity
☐	☐ Graph analysis
☐	☐ Multivariate modeling or predictive analysis

Functional and/or effective connectivity | *Report the measures of dependence used and the model details (e.g. Pearson correlation, partial correlation, mutual information).*

Graph analysis | *Report the dependent variable and connectivity measure, specifying weighted graph or binarized graph, subject- or group-level, and the global and/or node summaries used (e.g. clustering coefficient, efficiency, etc.).*

Multivariate modeling and predictive analysis | *Specify independent variables, features extraction and dimension reduction, model, training and evaluation metrics.*
